# Long non-coding RNA SNHG5 promotes the osteogenic differentiation of bone marrow mesenchymal stem cells via the miR-212-3p/GDF5/SMAD pathway

**DOI:** 10.1186/s13287-022-02781-8

**Published:** 2022-03-28

**Authors:** Yineng Han, Qiaolin Yang, Yiping Huang, Lingfei Jia, Yunfei Zheng, Weiran Li

**Affiliations:** 1 Department of Orthodontics, Peking University School and Hospital of Stomatology, 22 Zhongguancun Avenue South, Haidian District, Beijing, 100081 People’s Republic of China; 2 Department of Oral and Maxillofacial Surgery, Peking University School and Hospital of Stomatology, 22 Zhongguancun Avenue South, Haidian District, Beijing, 100081 People’s Republic of China; 3 Central Laboratory, Peking University School and Hospital of Stomatology, 22 Zhongguancun Avenue South, Haidian District, Beijing, 100081 People’s Republic of China

**Keywords:** SNHG5, Osteogenesis, YY1, miR-212-3p, GDF5

## Abstract

**Background:**

The treatment of bone loss has posed a challenge to clinicians for decades. Thus, it is of great significance to identify more effective methods for bone regeneration. However, the role and mechanisms of long non-coding RNA small nucleolar RNA host gene 5 (SNHG5) during osteogenic differentiation remain unclear.

**Methods:**

We investigated the function of SNHG5, Yin Yang 1 (YY1), miR-212-3p and growth differentiation factor 5 (GDF5) in osteogenic differentiation of human bone marrow mesenchymal stem cells (hBMSCs) in vitro and in vivo. Molecular mechanisms were clarified by chromatin immunoprecipitation assay and dual luciferase reporter assay.

**Results:**

We found SNHG5 expression was upregulated during osteogenesis of hBMSCs. Knockdown of SNHG5 in hBMSCs inhibited osteogenic differentiation while overexpression of SNHG5 promoted osteogenesis. Moreover, YY1 transcription factor directly bound to the promoter region of SNHG5 and regulated SNHG5 expression to promote osteogenesis. Dual luciferase reporter assay confirmed that SNHG5 acted as a miR-212-3p sponge and miR-212-3p directly targeted GDF5 and further activated Smad1/5/8 phosphorylation. miR-212-3p inhibited osteogenic differentiation, while GDF5 promoted osteogenic differentiation of hBMSCs. In addition, calvarial defect experiments showed knockdown of SNHG5 and GDF5 inhibited new bone formation in vivo.

**Conclusion:**

Our results demonstrated that the novel pathway YY1/SNHG5/miR-212-3p/GDF5/Smad regulates osteogenic differentiation of hBMSCs and may serve as a potential target for the treatment of bone loss.

**Supplementary Information:**

The online version contains supplementary material available at 10.1186/s13287-022-02781-8.

## Background

The treatment of bone loss has posed a challenge to clinicians for decades; thus, scholars are committed to identifying therapeutic approaches for bone regeneration [1]. Bone marrow mesenchymal stem cells (BMSCs), as widely used adult mesenchymal stem cells (MSCs), have garnered increasing attention. BMSCs with the potential to differentiate into osteoblasts and adipocytes in bone play important roles in maintaining bone homeostasis, and their dysfunction may lead to abnormal bone metabolism [2]. However, the molecular mechanisms of BMSCs during osteogenic differentiation are not fully understood. A better understanding of osteogenic differentiation in human BMSCs (hBMSCs) may provide novel strategies for bone regeneration.

As an important subset of non-coding RNAs (ncRNAs), long ncRNAs (lncRNAs) exceeding 200 nucleotides in length play important roles in various biological and pathological processes including osteogenesis [3] and the osteogenic differentiation of hBMSCs [4–6]. A recent study showed that lncRNA *MIR22HG* promotes osteogenic differentiation by downregulating phosphatase and tensin homolog, thereby activating AKT signaling [6]. However, a large number of lncRNAs and the underlying mechanisms during osteogenesis remain unknown. MicroRNAs (miRNAs) are another class of ncRNAs, usually 19–22 nucleotides in length. They bind to a partially complementary sequence in the 3′ untranslated region (UTR) of the target mRNA, inhibiting mRNA translation or destabilization [7]. One of the important functions of lncRNAs is that it can act as miRNA “sponges” by binding to miRNA-binding sites, forming a regulatory competing endogenous RNA (ceRNA) network, further regulating the targeted mRNAs [8, 9].

LncRNA small nucleolar RNA host gene 5 (SNHG5) is 524 base pairs (bp) in size and is located on chromosome 6q14 in humans. It is drawing increasing attention because of its role in a variety of cancers such as glioma, colorectal cancer, osteosarcoma and other malignancies [10–13]. Previous studies have reported that SNHG5 promoted osteogenic differentiation and apoptosis of hBMSCs via miR-582-5p/RUNX3 feedback loop [14]. SNHG5 suppresses the chondrogenic differentiation of human adipose-derived stem cells via targeting miR-23a-3p/ SOX6/SOX5 axis [15].

Yin Yang 1 (YY1) is a ubiquitously expressed transcription factor, belonging to the GLI-Krüppel family of zinc finger transcription factors. It regulates various genes associated with proliferation, differentiation, autophagy, and DNA repair [16]. Our previous sequencing results demonstrated that SNHG5 was upregulated during osteogenesis. However, it remains unclear whether it is important for hBMSCs osteogenic differentiation, and if so, what’s the underlying mechanism of its action? Therefore, in the present study, we aimed to investigate the critical role and molecular mechanisms of SNHG5 in the osteogenesis of hBMSCs.

## Materials and methods

### Cell culture

The primary culture of hBMSCs were obtained from ScienCell Research Laboratory (Carlsbad, CA, USA) and cells at passages 3–6 were used for this study. Cells were cultured in α-modified Eagle’s medium (α-MEM; Gibco, Grand island, NY, USA) supplemented with 10% fetal bovine serum (FBS; Gibco) and 1% penicillin/streptomycin (Gibco) as the proliferation medium (PM) as previously described [17]. Osteoinduction began when hBMSCs reached 70–80% confluence, and the osteogenic medium (OM) contained 10 mM β-glycerophosphate (Sigma-Aldrich, St. Louis, MO, USA), 100 nM dexamethasone (Sigma), and 200 μM L-ascorbic acid (Sigma). Human embryonic kidney (293 T) cells were obtained from the American Type Culture Collection (Manassas, VA, USA) and cultured in Dulbecco’s modified Eagle medium (DMEM) supplemented with 10% FBS (Gibco) and 1% penicillin/streptomycin (Gibco). All cells were cultured in a humid atmosphere with 95% air, 5% CO_2_ at 37 °C.

### Alkaline phosphatase staining (ALP) and activity

ALP staining and the ALP activity assay were performed as previously described [18]. Briefly, after 7 days of osteogenic induction, cells were washed with phosphate-buffered saline (PBS) and fixed in 4% paraformaldehyde. Then, the 5-bromo-4-chloro-3-indolyl-phosphate/Nitro-Blue-Tetrazolium Staining Kit (CoWin Biotech, Beijing, China) was used for ALP staining. ALP activity was quantified using the ALP Activity Kit (Biovision, Milpitas, CA, USA) and calculated after normalization to the total protein content.

### Alizarin red S staining (ARS) and quantification

ARS staining and quantification were performed as previously described [18]. After 14 days of osteogenic induction, cultured cells were fixed in 4% paraformaldehyde for 30 min at room temperature. After being washed with distilled water for three times, cells were stained with 0.1% ARS (pH 4.2; Sigma) solution for 20 min. The degree of mineralization was quantified by dissolving the stain in cetylpyridinium chloride (Sigma) for 1 h and detecting the absorbance of the collected solution at 570 nm. ARS intensity relative to the control treatment was normalized to the total protein content.

### RNA collection and quantitative reverse transcription-polymerase chain reaction (qRT-PCR)

TRIzol reagent (Invitrogen, Carlsbad, CA, USA) was used for total cellular RNA extraction, and a cDNA Reverse Transcription Kit (Takara, Tokyo, Japan) was used to synthesize cDNA from 2 µg total RNA. Then qRT-PCR was conducted as previously described [19] using SYBR Green Master Mix (Roche, Basel, Switzerland) and the ABI Prism 7500 Real-Time PCR System (Applied Biosystems, Foster City, CA, USA). Primers are listed in Additional file 1: Table S1. GAPDH was used as an endogenous normalization control for mRNAs and lncRNAs, and U6 for miRNAs. The 2^−ΔΔCt^ method was used to analyze the relative expression. Data information: Data are displayed as mean ± SD and show one representative of *n* ≥ 3 independent experiments with three biological replicates.

### Cell fractionation assays

The cell fractionation assays were performed using a Nuclei Isolation Kit (Invent Biotechnologies, Beijing, China) to separate cytoplasmic and nuclear RNA as previously described [20]. Briefly, cytoplasm extraction buffer was added to the harvested cells with vigorous vortexing for 15 s. After incubation on ice for 5 min, the mixtures were centrifuged at top speed in a microcentrifuge at 4 °C for 5 min. After centrifugation, RNA from the cytosol fraction (the supernatant) and nuclear fraction (the pellet) was isolated using TRIzol as described above. Metastasis-associated lung adenocarcinoma transcript 1 (MALAT1) and U6 served as fractionation indicators. Primers are listed in Additional file 1: Table S1.

### RNA oligoribonucleotides

RNA oligoribonucleotides including small interfering RNAs (siRNAs) targeting SNHG5 (si-SNHG5-1, si-SNHG5-2), YY1 (si-YY1-1, si-YY1-2, si-YY1-3), GDF5 (si-GDF5-1, si-GDF5-2) and their corresponding siRNA negative control (si-NC) were obtained from GenePharma (Suzhou, China). miR-212-3p mimic, miR-212-3p inhibitor, and the corresponding miRNA control were purchased from (RiboBio, Guangzhou, China). Sequences are listed in Additional file 1: Table S2.

### *siRNA transfection assay*s

siRNA transfection assays were performed using Lipofectamine 3000 (Invitrogen) as previously described [21]. Briefly, hBMSCs at a confluence of 80% were transfected with siRNAs to knockdown the expression of SNHG5, YY1, or GDF5.

### Lentivirus infection

Recombinant lentiviruses containing full-length SNHG5 (SNHG5-OE), YY1 (YY1-OE), and negative control vectors (NC-OE) were purchased from GenePharma Co. (Suzhou, China). Lentivirus infection of hBMSCs was performed as previously described at a multiplicity of infection of 100 [22]. Infection was performed by exposing hBMSCs to dilutions of viral supernatant in the presence of polybrene (5 mg/mL) and fresh medium for 24 h, followed by selection with puromycin (Sigma) at 1 mg/mL. The percentage of cells positive for green fluorescent protein was used to determine the transduction efficiency observed under an inverted fluorescence microscope (TE2000-U; Nikon, Tokyo, Japan).

### RNA sequencing

After 7 days of osteoinduction, total RNA samples isolated from hBMSCs transfected with si-SNHG5 or si-NC were used for sequencing and analyses as previously described [22]. cDNA was synthesized and PCR was performed to amplify the library DNA. The final libraries were quantified using the KAPA Library Quantification Kit (KAPA Biosystems, Cape Town, South Africa) and the Agilent 2100 Bioanalyzer. After qRT-PCR validation, the libraries were subjected to paired-end sequencing with 150 bp read lengths on the Illumina NovaSeq sequencer (Illumina, San Diego, CA, USA). Differentially expressed genes (DEGs) were defined as fold change ≥ 2 and *p* < 0.05. DEG annotation was performed based on the information obtained from the ENSEMBL, NCBI, Uniprot, and Kyoto encyclopedia of genes and genomes (KEGG) databases. Gene ontology (GO) classification was visualized by DAVID v6.8 (http://david.ncifcrf.gov) webserver.

### miRNA sequencing

Total RNA samples isolated from hBMSCs transfected with si-SNHG5 or si-NC were used for subsequent sequencing and analyses as previously described [23]. Small RNA sequencing libraries were created following the Illumina TruSeq Small RNA Sample Preparation protocol. Small RNA libraries were pooled and sequenced for each cDNA molecule with the Illumina Hiseq 2500 sequencer (Illumina). The conserved miRNAs were identified by comparing the sRNA reads with known miRNAs collected from the miRBase (http://www.mirbase.org/). Novel miRNAs were identified from the unmatched reads. The putative miRNA precursors were identified by miRDeep2. Only those with precursors found in the genome were identified as conserved or novel miRNAs. The potential miRNA targets were predicted using miRanda (http://www.miranda.org/). Differential expression analyses were performed using the edgeR and limma packages. Functional annotation of the target genes and enrichment analyses of the DEG miRNA were performed with KOBAS3.0.

### *Fluorescence *in situ* hybridization (FISH)*

FISH assay was performed using the FISH Kit (RiboBio) as previously described [24]. Briefly, cells were rinsed with PBS and fixed in 4% paraformaldehyde for 15 min at room temperature. Then, the cells were permeabilized in 0.5% Triton X-100 at 4 °C for 5 min and washed three times with PBS. Next, the samples were pre-hybridized at 55 °C for 30 min. For hybridization, anti-SNHG5 probes were added to the hybridization solution and cells were incubated at 37 °C overnight in the dark. Then, the cells were counterstained with DAPI (Servicebio, Wuhan, China), and images were taken using a confocal laser scanning microscope (Carl Zeiss, Oberkochen, Germany).

### Western blotting analyses

For protein extraction, cells were harvested, washed, and lysed in radioimmunoprecipitation assay buffer supplemented with a protease inhibitor mixture (Roche) as previously described [17]. The Pierce BCA Protein Assay Kit (Thermo Fisher Scientific) was used to determine the protein concentration. Equal amounts of proteins were separated on sodium dodecyl sulfate–polyacrylamide gel electrophoresis gels and electroblotted onto polyvinylidene fluoride membranes (Bio-Rad, Hercules, CA, USA). After blocking in 5% milk for 1 h, the membranes were incubated overnight at 4 °C with primary antibodies against osteocalcin (OCN, 1:1000; Abcam, Cambridge, UK), runt-related transcription factor 2 (RUNX2, 1:1000; Abcam), YY1 (1:1000; Cell Signaling Technology, Beverly, MA, USA), GDF5 (1:1000; Abcam), phosphorylated Smad1/5/8 (p-Smad1/5/8, 1:1000; Cell Signaling Technology), Smad1/5/8 (1:1000; Abcam), and β-actin (1:1000; Zhongshan Goldenbridge, Beijing, China). The next day, the membranes were incubated with secondary antibodies (1:10,000; Zhongshan Goldenbridge) at room temperature for 1 h after washing with TBST for three times. The Enhanced Chemiluminescence Kit (Applygen, Beijing, China) was used to visualize the protein bands, which were quantified with ImageJ software (http://rsb.info.nih.gov/ij/). The signals of target bands were normalized to β-actin band relative to the control groups.

### Immunofluorescence staining of RUNX2

Immunofluorescence staining of RUNX2 was performed as previously described [25, 26]. Briefly, cells were fixed in 4% paraformaldehyde and then permeabilized with 0.1% Triton X-100 for 10 min at room temperature. Then, the cells were incubated with 5% normal goat serum for 40 min at room temperature and incubated with primary antibody against RUNX2 (1:200; Abcam) at 4 °C overnight. Next, they were incubated with secondary antibody in the dark at room temperature for 1 h. DAPI (Servicebio) was used to counterstain the nucleus. Staining was visualized using a confocal laser scanning microscope (Carl Zeiss).

### Dual luciferase reporter assay

Dual luciferase assays were performed in both 293T and hBMSCs as previously described [18]. Briefly, cells were seeded in 48-well plates, and a mixture of 40 ng luciferase reporter, 4 ng Renilla, and 100 nM miR-212-3p mimic or control was transfected into cells with Lipofectamine 3000 (Invitrogen). After 24 h, luciferase activity was determined using the Dual-Luciferase Reporter Assay System (Promega, Beijing, China) normalized to Renilla luciferase.

### Flow cytometry

Flow cytometry was performed to identify MSCs as previously described [27]. Cells in each group were detached using trypsin and washed with phosphate buffer saline (PBS), then incubated at 4 °C for 30 min in the dark. Fluorescein isothiocyanate (FITC) was bound to antibodies CD34, CD45, CD105, CD90 and CD29 (eBiolegend, San Diego, CA, USA). Labeling was quantified using the BD Accuri C6 Flow Cytometer (USA). Homotypically matched normal immunoglobulin G (IgG) antibody was used as a control. Data were analyzed with the FlowJo software packages (TreeStar, USA).

### In vivo bone formation assay

Animal experiments were approved by the Peking University Animal Care and Use Committee (LA2018305). In vivo bone formation assay was performed as previously described [18]. Briefly, hBMSCs transfected with si-SNHG5, si-GDF5, si-NC, SNHG5-OE, NC-OE were cultured with OM for 7 days. We used polylactic-co-glycolic acid (PLGA; Melone, Dalian, China) scaffolds prepared as thin circular slices (Φ = 4 mm, thickness of 2 mm) as a scaffold material. The cells were seeded on the surface of the scaffold material at a density of 5 × 105 cells in each group and incubated at 37 °C for 1 h before transplantation. Male nude mice (5-week-old, weighing nearly 17 g, 5 mice/group) were purchased from Vital River Laboratory Animal Technology Co. (Beijing, China), and the operation was performed under general anesthesia. As previously described [21], we first constructed critical calvarial defect model using a dental drill. Then, we removed the pericranium and gently transplanted the seeded scaffolds into the defects. Finally, 5–0 Vicryl sutures were used to close the skin incision. After 5 weeks, the skulls were harvested and fixed in 4% paraformaldehyde for 24 h for further observation.

### Micro-computed tomography (micro-CT) analyses

Micro-CT analyses of specimens were conducted as previously described [18]. Before micro-CT analyses, the skull specimens were collected and fixed in 4% paraformaldehyde for 1 day. Then. micro-CT images were obtained from high-resolution Inveon micro-CT (Siemens, Munich, Germany) with a resolution of 8.99 μm at 80 kV, 500 μA and an exposure time of 1500 ms. All specimens were scanned using the uniform parameters in the same container. Multimodal three-dimensional (3D) visualization software (Inveon Research Workplace; Siemens, Munich, Germany) was used to reconstruct 3D images. Bone volume (BV) and bone volume/tissue volume (BV/TV) were calculated.

### Hematoxylin and eosin staining (H&E), Masson’s trichrome staining, and immunohistochemical analyses

The skull specimens were decalcified in 10% ethylene diamine tetraacetic acid (pH 7.4) for 14 days and then washed, dehydrated, and embedded in paraffin. Sections (5 μm thickness) were stained with H&E and Masson’s trichrome as previously described [21]. For immunohistochemical staining, the sections were blocked in 3% goat serum albumin (Zhongshan Goldenbridge) and then incubated overnight at 4 °C with primary antibody against OCN (1:200; Abcam). Then, the sections were incubated with the corresponding secondary antibodies. All the images were captured under a light microscope (Olympus, Tokyo, Japan).

### Chromatin immunoprecipitation assay

Chromatin immunoprecipitation (ChIP) assays were performed using the EZ-Magna ChIP Assay Kit (Merck Millipore, Darmstadt, Germany) as previously described [20]. Briefly, hBMSCs were first crosslinked with 1% formaldehyde, collected, lysed, and sonicated to shear DNA. Then the DNA–protein complexes were isolated with antibodies against YY1 (Cell Signaling Technology) and normal rabbit IgG (Merck Millipore) as a negative control at 4 °C overnight. The next day, the protein-DNA complexes were eluted and de-crosslinked. The purified DNA was quantified by qRT-PCR and relative enrichment was calculated as the amount of amplified DNA relative to values obtained from immunoprecipitation using normal IgG. Primers are listed in Additional file 1: Table S3.

### Statistical analyses

SPSS version 19.0 (IBM, Chicago, IL, USA) was used for statistical analyses. All data are expressed as the mean ± standard deviation with three biological replicates. The two-tailed unpaired Student’s *t* test was used for two-group comparisons. One-way analysis of variance was used for multiple group comparisons. The threshold of statistical significance was set at *p* < 0.05.

## Results

### SNHG5 promotes the osteogenic differentiation of hBMSCs

**Fig. 1:**
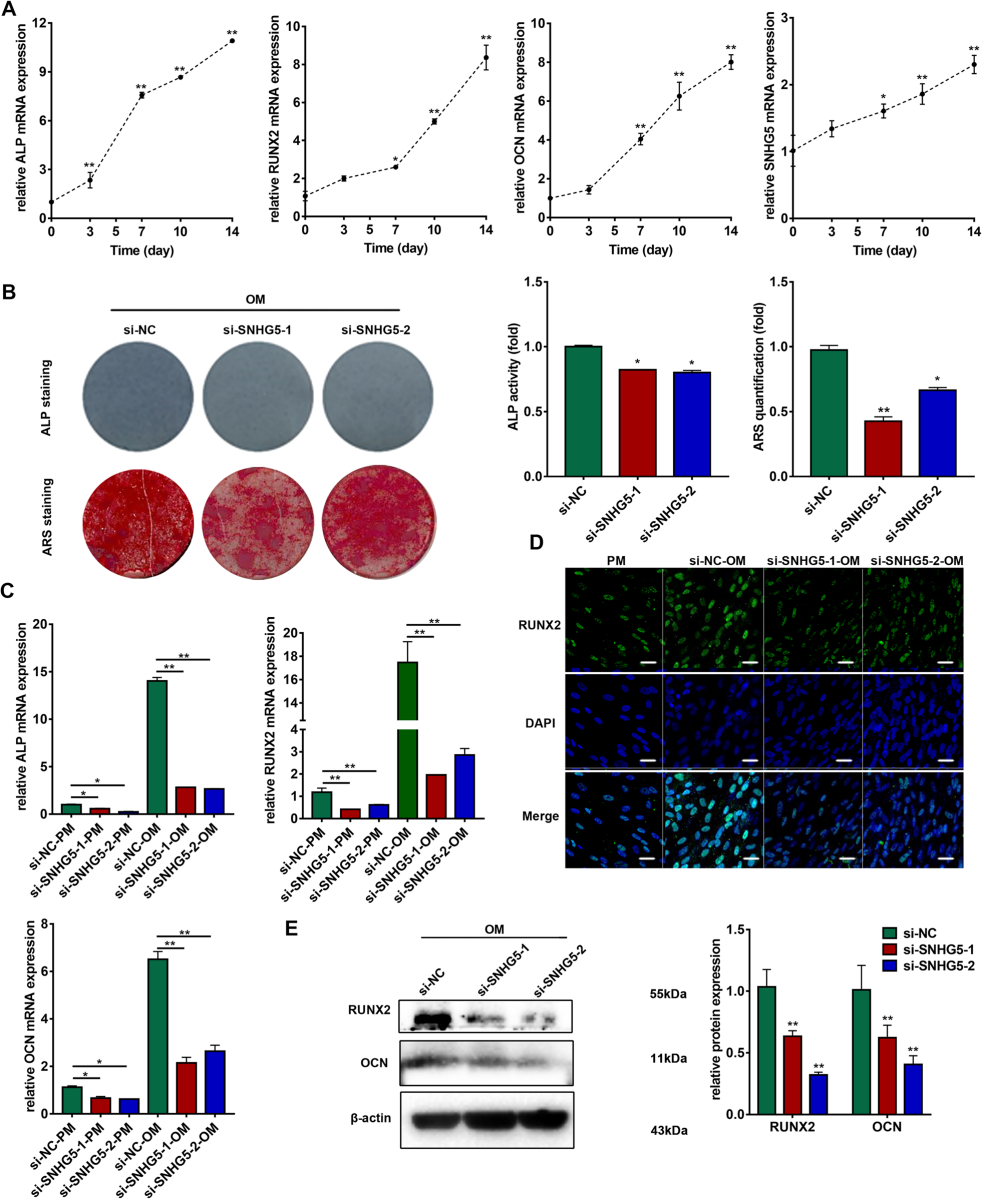
SNHG5 knockdown inhibits the osteogenic differentiation of hBMSCs. **a** Relative expression of ALP, RUNX2, OCN, and SNHG5 during osteoinduction of hBMSCs via qRT-PCR (*n* = 3). GAPDH was used for normalization relative to the day 0 group. **b** Images of ALP staining on day 7 of osteogenic differentiation, and ARS staining on day 14 of osteogenic differentiation in the si-NC, si-SNHG5-1, and si-SNHG5-2 groups (*n* = 3). Histograms show ALP activity and ARS staining quantification by spectrophotometry. **c** Relative mRNA expression of ALP, RUNX2, and OCN measured via qRT-PCR in the PM and OM on day 7. GAPDH was used for normalization (*n* = 3). **d** Confocal microscopy of RUNX2 with DAPI counterstaining of the si-NC, si-SNHG5-1, and si-SNHG5-2 groups after osteogenic induction for 7 days (*n* = 3). Scale bars: 20 μm. **e** Western blotting analyses of the protein expression of RUNX2, OCN, and β-actin in the si-NC, si-SNHG5-1, and si-SNHG5-2 groups after osteogenic induction for 7 days (*n* = 3). Histograms show the quantification of band intensities. β-actin was used for normalization relative to the si-NC group. (*p < 0.05; **p < 0.01). ALP, alkaline phosphatase; ARS, alizarin red S; GAPDH, glyceraldehyde-3-phosphate dehydrogenase; hBMSCs, human bone marrow mesenchymal stem cells; PM, proliferation medium; OCN, osteocalcin; OM, osteogenic medium; qRT-PCR, quantitative reverse transcription-polymerase chain reaction; RUNX2, runt-related transcription factor 2; SNHG5, small nucleolar RNA host gene 5

hBMSCs were first characterized by flow cytometry and the results showed that the hBMSCs expressed surface markers of human mesenchymal stem cells (CD90, CD105 and CD29), but not those of hematopoietic stem cells (CD34 and CD45) (Additional file 1: Fig. S1). Then, hBMSCs were used for osteogenic induction. qRT-PCR results showed that the mRNA levels of the three osteogenic markers ALP, RUNX2, and OCN were significantly upregulated, indicating the successful induction of hBMSCs into the osteogenic lineage (Fig. 1a). The expression of SNHG5 was significantly upregulated during osteogenic differentiation (Fig. 1a), indicating SNHG5 is involved in osteogenesis. To further determine the role of SNHG5 during the osteogenic differentiation of hBMSCs, lentivirus was transduced into hBMSCs to overexpress SNHG5 and siRNA targeting SNHG5 was used to knockdown SNHG5. Two siRNA (si-SNHG5-1, si-SNHG5-2) sequences targeting SNHG5 were used and the transfection effects were detected by qRT-PCR (Additional file 1: Fig. S2A). The lentivirus transduction efficiency of hBMSCs was confirmed by qRT-PCR and fluorescent staining (Additional file 1: Fig. S2B). After 7 days of osteogenic induction, ALP staining and activity were significantly decreased by SNHG5 knockdown (Fig. 1b). After 14 days of osteogenic induction, extracellular matrix mineralization, as revealed by ARS staining, decreased after SNHG5 knockdown (Fig. 1b). We also measured the mRNA expression of osteogenic markers by qRT-PCR in both PM and OM for 7 days. SNHG5 knockdown significantly decreased the expression of ALP, RUNX2, and OCN after 7 days of osteogenic induction (Fig. 1c). Immunofluorescence staining results indicated that the protein level of RUNX2 was reduced in SNHG5 knockdown cells (Fig. 1d). The downregulation of RUNX2 and OCN by SNHG5 knockdown after 7 days of osteogenic induction was also confirmed by western blotting analyses (Fig. 1e). On the other hand, overexpression of SNHG5 significantly enhanced ALP staining and ALP activity (Additional file 1: Fig. S2C). In addition, ARS staining showed SNHG5 overexpression promoted more extracellular matrix mineralization (Additional file 1: Fig. S2C) and elevated gene expression of osteogenic markers in both PM and OM for 7 days (Additional file 1: Fig. S2D). Immunofluorescence staining results also indicated the enhanced expression of RUNX2 in SNHG5 overexpressing cells compared to the control group (Additional file 1: Fig. S2E). The protein levels of RUNX2 and OCN were also upregulated in SNHG5 overexpressing cells after 7 days of osteogenic induction (Additional file 1: Fig. S2F).

### Transcription factor YY1 regulates SNHG5 expression

**Fig. 2:**
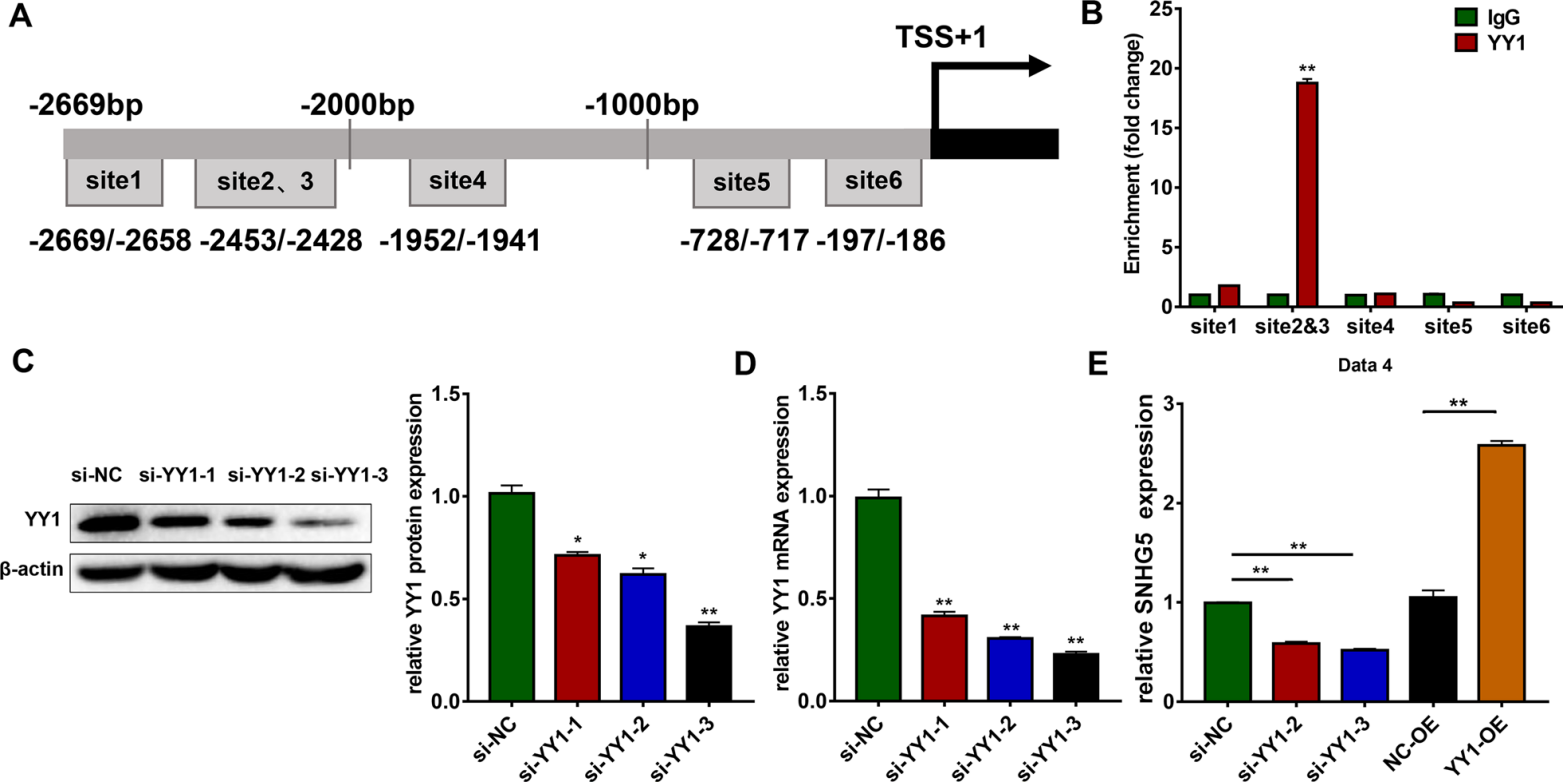
YY1 induces SNHG5 expression. **a** Diagram of the SNHG5 promoter and location of the primers. Positions marked are relative to the TSS. **b** ChIP-qRT-PCR show the interaction between YY1 and the SNHG5 promoter in hBMSCs (*n* = 3). IgG was used for normalization. **c** The efficiency of transient transfection of si-YY1-1, si-YY1-2, and si-YY1-3 by western blotting (*n* = 3). Histograms show the quantification of band intensities. β-actin was used for normalization relative to the si-NC group. **d** The efficiency of transient transfection of si-YY1-1, si-YY1-2, and si-YY1-3 by qRT-PCR (*n* = 3). GAPDH was used for normalization relative to the si-NC group. **e** Relative expression of SNHG5 after downregulation or upregulation of YY1 by qRT-PCR (*n* = 3). GAPDH was used for normalization. (*p < 0.05; **p < 0.01). ChIP, Chromatin immunoprecipitation; GAPDH, glyceraldehyde-3-phosphate dehydrogenase; hBMSCs, human bone marrow mesenchymal stem cells; qRT-PCR, quantitative reverse transcription-polymerase chain reaction; TSS, transcriptional start site; SNHG5, small nucleolar RNA host gene 5; YY1, Yin Yang 1

To further explore the regulatory mechanisms of SNHG5 during osteogenesis, the regulatory elements of the SNHG5 promoter were analyzed using the web-based transcription factor search site (http://jaspar.binf.ku.dk/) in the region 3000 bp upstream from the transcriptional start site (TSS) of SNHG5. The top six putative YY1-binding sites were chosen for further analyses (sites 1–6). ChIP assays were performed with the antibody against YY1 and five pairs of primers flanking the regions of the predicted YY1-binding sites (as sites 2 and 3 were too close to design primers separately, we designed only one pair of primers for these regions) (Fig. 2a). The results showed that only the fragment containing binding sites 2 and 3 were responsible for the binding of YY1 to the SNHG5 promoter region (Fig. 2b), indicating that the vicinity of the 2000 bp region upstream of the SNHG5 TSS contains most of the binding elements of YY1. To further explore the regulation relationship between SNHG5 and YY1, we first used siRNA targeting YY1 (si-YY1-1, si-YY1-2, si-YY1-3) to knockdown YY1. Both western blotting and qRT-PCR results demonstrated that YY1 was successfully knocked down (Fig. 2c, d), especially si-YY1-2 and si-YY1-3. Therefore, we chose si-YY1-2 and si-YY1-3 for further experiments. We used lentivirus to overexpress YY1 in the hBMSCs. The efficiency of lentivirus transduction was detected by qRT-PCR and western blotting (Additional file 1: Fig. S3A, B). Knockdown of YY1 inhibited SNHG5 transcription in hBMSCs, whereas overexpression of YY1 increased the expression of SNHG5 (Fig. 2e). These results indicate that YY1 can bind to the SNHG5 promoter to activate its transcription.

### YY1 promotes the osteogenic differentiation of hBMSCs

**Fig. 3:**
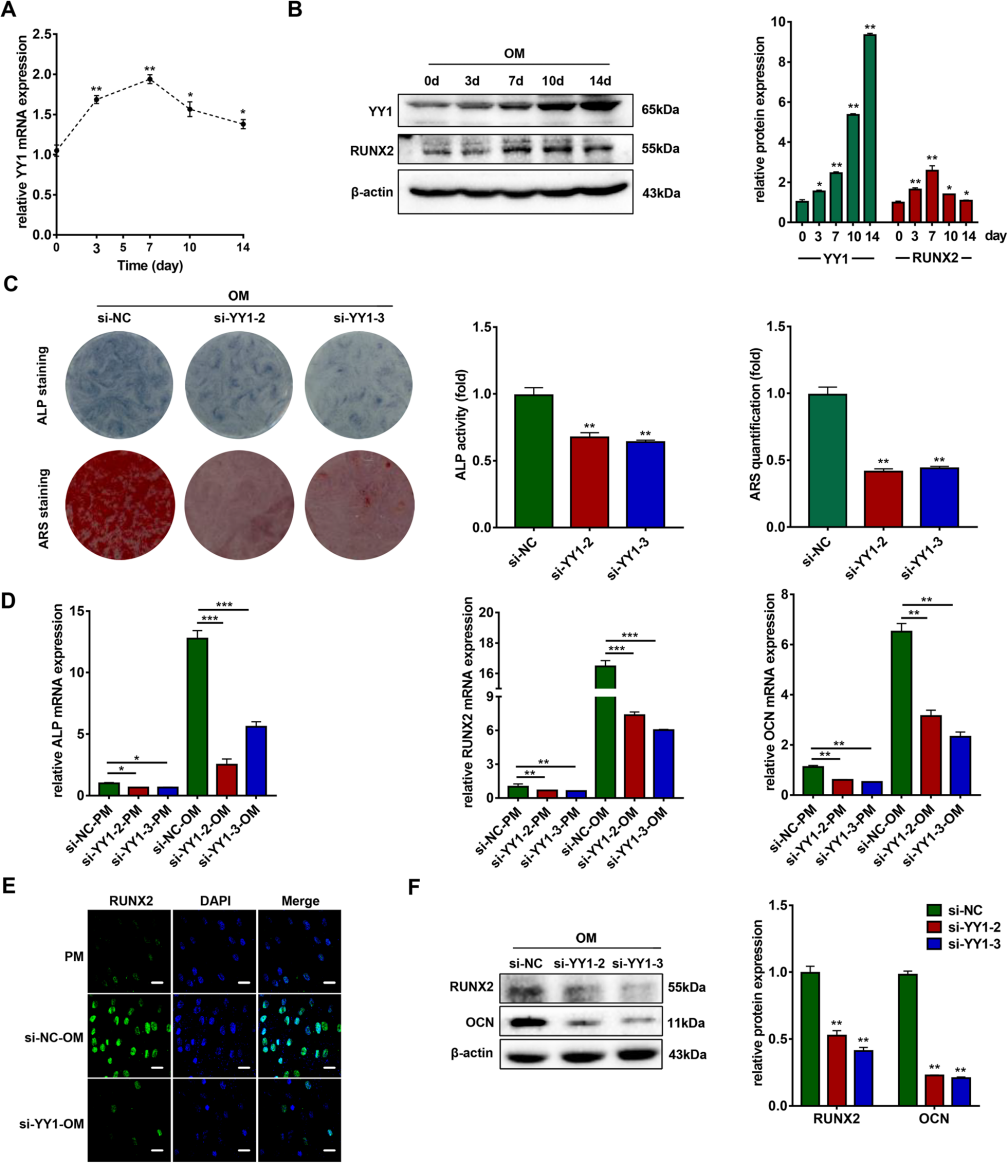
YY1 knockdown inhibits the osteogenic differentiation of hBMSCs. **a** Relative mRNA expression of YY1 during the osteoinduction of hBMSCs (*n* = 3). GAPDH was used for normalization relative to the day 0 group. **b** Protein expression of YY1 and the internal control β-actin during osteoinduction of hBMSCs (*n* = 3). Histograms show the quantification of band intensities. β-actin was used for normalization relative to the day 0 group. **c** Images of ALP staining after 7 days of osteogenic differentiation, and ARS staining after 14 days of osteogenic differentiation in the si-NC, si-YY1-2, and si-YY1-3 groups (*n* = 3). Histograms show ALP activity and ARS staining quantification by spectrophotometry. **d** Relative mRNA expression of ALP, RUNX2, and OCN measured via qRT-PCR in PM and OM on day 7 (*n* = 3). GAPDH was used for normalization. **e** Confocal microscopy of RUNX2 with DAPI counterstaining of the si-NC and si-YY1 groups after osteogenic induction for 7 days (*n* = 3). Scale bars: 20 μm. **f** Western blotting analyses of the protein expression of RUNX2, OCN, and β-actin in the si-NC, si-YY1-2, and si-YY1-3 groups after osteogenic induction for 7 days (*n* = 3). Histograms show the quantification of band intensities. β-actin was used for normalization relative to the si-NC group (*p < 0.05; **p < 0.01; ***p < 0.001). ALP, alkaline phosphatase; ARS, alizarin red S; GAPDH, glyceraldehyde-3-phosphate dehydrogenase; hBMSCs, human bone marrow mesenchymal stem cells; PM, proliferation medium; OCN, osteocalcin; OM, osteogenic medium; qRT-PCR, quantitative reverse transcription-polymerase chain reaction; RUNX2, runt-related transcription factor 2; YY1, Yin Yang 1

Then, we explored the function of YY1 during osteogenic differentiation. With the osteogenic induction of hBMSCs, both the mRNA and protein expression of YY1 were increased (Fig. 3a, b). ALP staining and activity showed that knockdown of YY1 inhibited osteogenic differentiation of hBMSCs for 7 days (Fig. 3c). ARS staining and quantification after 14 days of osteogenic differentiation displayed similar results as those of ALP staining and activity (Fig. 3c). Knockdown of YY1 decreased the mRNA expression levels of ALP, RUNX2, and OCN on day 7 in hBMSCs in both PM and OM (Fig. 3d). Immunofluorescence staining results indicated the protein level of RUNX2 were downregulated in the YY1 knockdown group compared to the control group (Fig. 3e). Moreover, the relative protein expression of RUNX2 and OCN was decreased in YY1 knockdown cells cultured in OM for 7 days (Fig. 3f). In addition, ALP staining and activity were increased in the YY1 overexpression group after 7 days of osteogenic induction (Additional file 1: Fig. S3C). A similar trend in ARS staining was detected after osteoinduction for 14 days (Additional file 1: Fig. S3C). The qRT-PCR analyses confirmed that overexpression of YY1 increased the mRNA expression of ALP, RUNX2, and OCN (Additional file 1: Fig. S3D). Immunofluorescence staining results showed a higher level of RUNX2 expression in SNHG5 overexpressing hBMSCs after 7 days of osteogenic differentiation (Additional file 1: Fig. S3E). The protein levels of RUNX2 and OCN were also increased when YY1 was overexpressed, consistent with the immunofluorescence staining results (Additional file 1: Fig. S3F).

### SNHG5 serves as a sponge for miR-212-3p

**Fig. 4:**
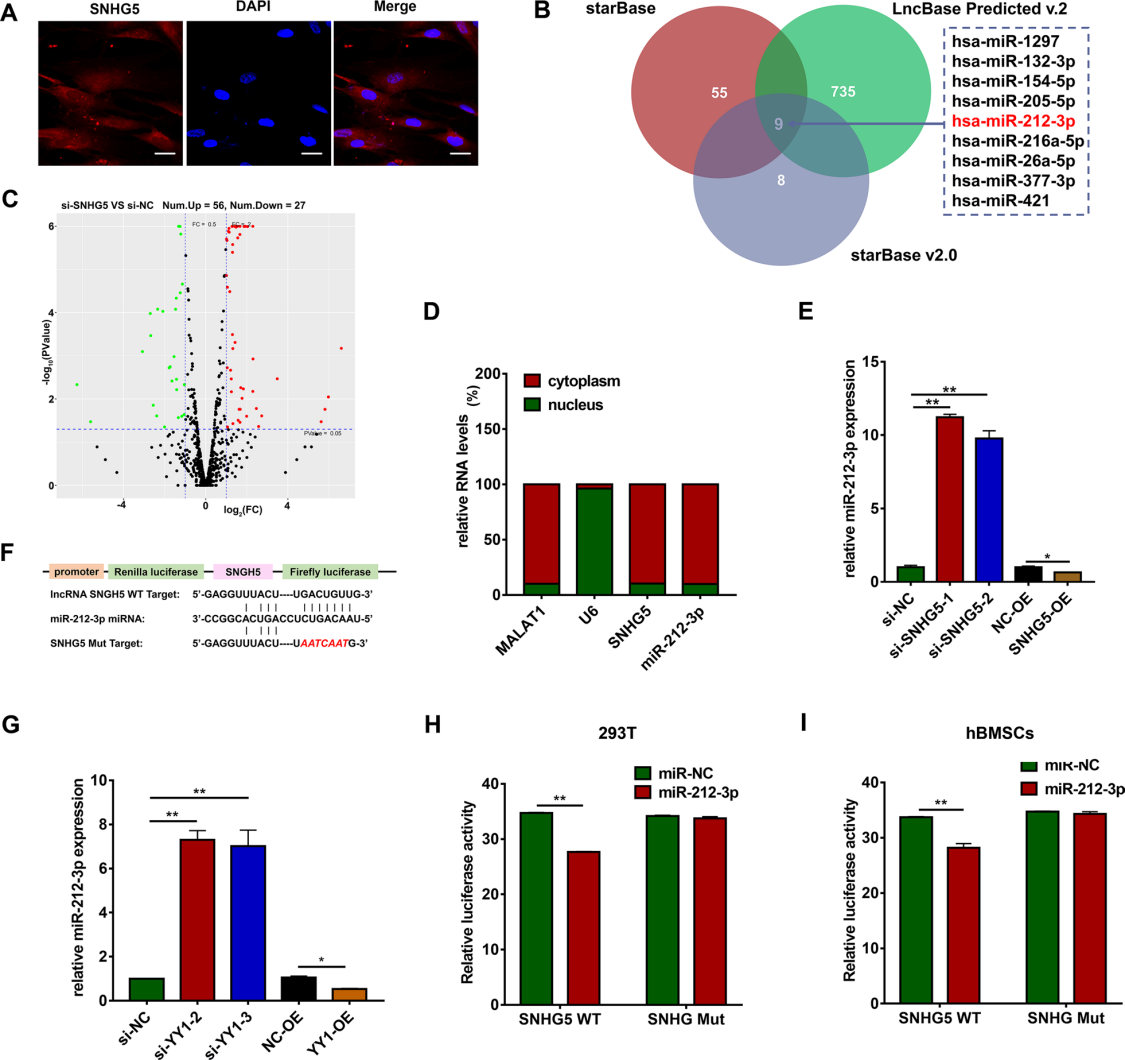
SNHG5 serves as a sponge for miR-212-3p. **a** Confocal FISH images show the subcellular localization of SNHG5 in hBMSCs (*n* = 3). Nuclei were stained by DAPI (blue). Scale bars: 20 μm. **b** Venn diagrams show the number of potential miRNAs targeting SNHG5 (including miR-212-3p). The potential miRNAs were predicted by three databases: LncBase Predicted v.2, starBase, starBase v2.0. p. **c** Volcano plot shows that a total of 56 genes were significantly upregulated and 27 genes were significantly downregulated in SNHG5 knockdown hBMSCs. The DEGs were defined as fold change ≥ 2 and *p* < 0.05. **d** Percentage of nuclear and cytoplasmic RNA levels of SNHG5 and miR-212-3p (*n* = 3). MALAT1 and U6 were used as fractionation indicators. **e**, **g** Relative RNA expression of miR-212-3p after downregulation or upregulation of SNHG5 or YY1 by qRT-PCR (*n* = 3). U6 was used for normalization. **f** Schematic diagram of SNHG5 WT and SNHG5 Mut luciferase reporter plasmids. A putative miR-212-3p target site of SNGH5 was predicted in bioinformatics analyses. **h**, **i** Dual luciferase reporter assays validate the interaction between miR-212-3p and SNHG5 in 293 T cells and hBMSCs (*n* = 3). (*p < 0.05; **p < 0.01; ***p < 0.001). DEGs, differentially expressed genes; FISH, fluorescence in situ hybridization; GAPDH, glyceraldehyde-3-phosphate dehydrogenase; hBMSC, human bone marrow mesenchymal stem cells; MALAT1, metastasis-associated lung adenocarcinoma transcript 1; qRT-PCR, quantitative reverse transcription-polymerase chain reaction; SNHG5, small nucleolar RNA host gene 5

The full-length sequence and RNA secondary structure information of SNHG5 are shown in Additional file 1: Fig. S4. FISH assay demonstrated that SNHG5 was mainly expressed in the cytoplasm of hBMSCs (Fig. 4a), suggesting that SNHG5 may serve as a ceRNA by binding to miRNAs and further competing with mRNAs. Both miRNA sequencing and three databases (starBaseV2, http://starbase.sysu.edu.cn/starbase2/index.php), LncBase Predicted v.2 (http://carolina.imis.athena-innovation.gr/diana_tools/web/index.php?r=lncbasev2%2Findex-predicted), starBase (http://starbase.sysu.edu.cn/) predicted that miR-212-3p had a high probability of binding to SNHG5 (Fig. 4b, c). Cell fractionation assays further verified our hypothesis that both SNHG5 and miR-212-3p were mainly localized in the cytoplasm, indicating their possible interaction (Fig. 4d). We also found that miR-212-3p expression was significantly downregulated following SNHG5 overexpression and upregulated after SNHG5 or YY1 knockdown (Fig. 4e, g). Next, we explored whether miR-212-3p could directly bind to the SNHG5 target site. Luciferase reporters containing wild-type (SNHG5 WT) or mutant (SNHG5 Mut) SNHG5 were constructed (Fig. 4f). The results demonstrated that SNHG5 WT reporter activity was markedly inhibited by miR-212-3p in 293 T cells and hBMSCs, while SNHG5 Mut was not affected (Fig. 4h, i).

### miR-212-3p inhibits the osteogenic differentiation of hBMSCs

**Fig. 5:**
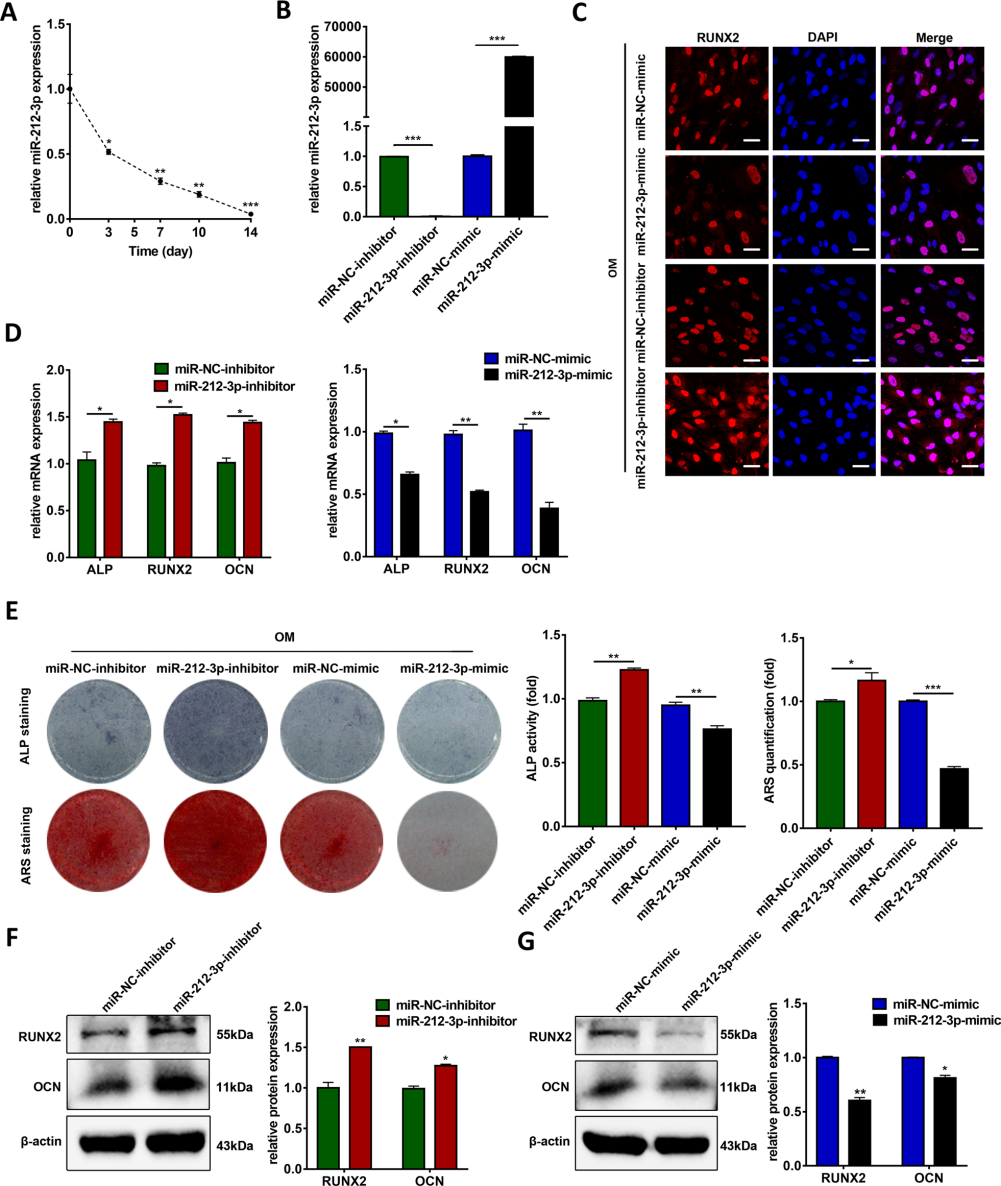
miR-212-3p inhibits osteogenic differentiation of hBMSCs. **a** Relative expression of miR-212-3p during osteoinduction of hBMSCs (*n* = 3). GAPDH was used for normalization relative to the day 0 group. **b** Transfection efficiency of miR-212-3p mimic or inhibitor by qRT-PCR in hBMSCs (*n* = 3). U6 was used for normalization. **c** Confocal microscopy of RUNX2 with DAPI counterstaining in the miR-NC-inhibitor, miR-212-3p-inhibitor, miR-NC-mimic, miR-212-3p-mimic groups after osteogenic induction for 7 days (*n* = 3). Scale bars: 20 μm. **d** Relative mRNA expression of ALP, RUNX2, and OCN measured via qRT-PCR in PM conditions (*n* = 3). GAPDH was used for normalization. **e** Images of ALP staining after 7 days of osteogenic differentiation, and ARS staining after 14 days of osteogenic differentiation in the miR-NC-inhibitor, miR-212-3p-inhibitor, miR-NC-mimic, miR-212-3p-mimic groups (*n* = 3). Histograms show ALP activity and AZR staining quantification by spectrophotometry. **f**, **g** Western blotting analyses of the protein expression of RUNX2, OCN, and β-actin in the miR-NC-inhibitor, miR-212-3p-inhibitor and miR-NC-mimic, miR-212-3p-mimic groups after osteogenic induction for 7 days (*n* = 3). Histograms show the quantification of band intensities. β-actin was used for normalization. (*p < 0.05; **p < 0.01; ***p < 0.001). ALP, alkaline phosphatase; GAPDH, glyceraldehyde-3-phosphate dehydrogenase; GDF5, growth differentiation factor 5; hBMSCs, human bone marrow mesenchymal stem cells; PM, proliferation medium; OCN, osteocalcin; OM, osteogenic medium; qRT-PCR, quantitative reverse transcription-polymerase chain reaction; RUNX2, runt-related transcription factor 2

Then, we investigated the function of miR-212-3p during osteogenic differentiation. With the osteogenic induction of hBMSCs, miR-212-3p was noticeably downregulated (Fig. 5a). qRT-PCR results confirmed that miR-212-3p-inhibitor successfully downregulated the expression of miR-212-3p, while miR-212-3p-mimic markedly upregulated the expression of miR-212-3p in hBMSCs (Fig. 5b). Immunofluorescence staining results indicated the upregulated protein level of RUNX2 in the miR-212-3p knockdown group and downregulated protein level of RUNX2 in the miR-212-3p overexpression group compared to the control groups (Fig. 5c). Knockdown of miR-212-3p enhanced the mRNA expression levels of ALP, RUNX2, and OCN, while overexpression of miR-212-3p decreased the expression of these osteogenic genes (Fig. 5d). ALP staining and activity results showed that knockdown of miR-212-3p promoted osteogenic differentiation, while overexpression of miR-212-3p inhibited osteogenic differentiation of hBMSCs for 7 days (Fig. 5e). ARS staining and quantification after 14 days of osteogenic differentiation showed similar results as those of ALP staining and activity (Fig. 5e). Moreover, the relative protein expression of RUNX2 and OCN was enhanced in the miR-212-3p knockdown group, while decreased in the miR-212-3p overexpression group after osteogenic differentiation for 7 days (Fig. 5f, g).

### GDF5 is a target of SNHG5/miR-212-3p axis

To further elucidate the molecular mechanisms by which miR-212-3p regulated osteogenic differentiation in hBMSCs, we first used the miRWalk (http://mirwalk.umm.uni-heidelberg.de/), TargetScan (http://www.targetscan.org/) and miRDB (http://mirdb.org/) databases to predict the potential targets of miR-212-3p. The results showed that the 3′ UTR of GDF5 contained a miR-212-3p-binding site (Additional file 1: Fig. S5A). Moreover, we established SNHG5 knockdown hBMSCs and conducted transcriptome microarray analyses after osteoinduction for 7 days. A volcano plot showed knockdown of SNHG5 after 7 days of osteogenic induction, resulting in upregulation and downregulation of genes (Additional file 1: Fig. S5B), and GDF5 was one of the downregulated genes. Therefore, we chose GDF5 to further verify our hypothesis. GDF5 is a member of the transforming growth factor beta (TGF-β) superfamily, also named as bone morphogenetic protein (BMP) 14. Then, we cloned GDF5 3′ UTR and its mutant containing putative miR-212-3p-binding sites into the downstream luciferase reporter (Additional file 1: Fig. S5C). Compared to the control group, luciferase reporter activity was significantly reduced when miR-212-3p was overexpressed, and this reduction was relieved in mutated GDF5 3′ UTR in both 293T cells and hBMSCs (Additional file 1: Fig. S5D). Both GDF5 mRNA and protein expression was decreased after transfection of miR-212-3p mimic and increased following transfection of miR-212-3p inhibitors in hBMSCs compared to the control groups (Additional file 1: Fig. S5E, F).

### GDF5 promotes the osteogenic differentiation of hBMSCs

**Fig. 6:**
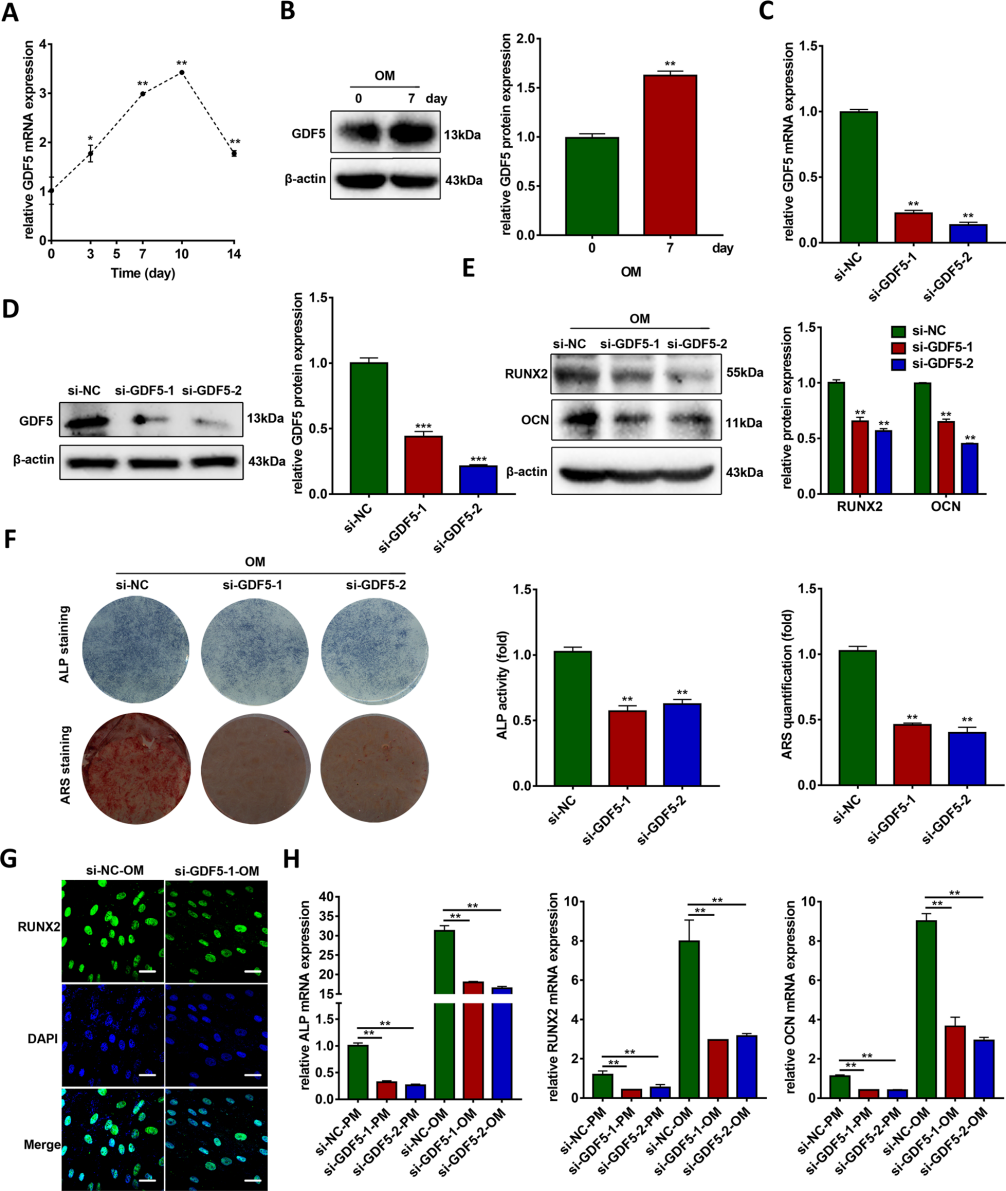
GDF5 knockdown inhibits osteogenic differentiation of hBMSCs. **a** Relative mRNA expression of GDF5 during osteoinduction of hBMSCs (*n* = 3). GAPDH was used for normalization relative to the day 0 group. **b** Protein expression of GDF5 and β-actin during osteoinduction of hBMSCs (*n* = 3). Histograms show the quantification of band intensities. β-actin was used for normalization relative to the day 0 group. **c** The efficiency of transient transfection of si-GDF5-1 and si-GDF5-2 by qRT-PCR. GAPDH was used for normalization relative to the si-NC group (*n* = 3). **d** The efficiency of transient transfection of si-GDF5-1 and si-GDF5-2 by western blotting analyses. Histograms show the quantification of band intensities (*n* = 3). β-actin was used for normalization relative to the si-NC group. **e** Western blotting analyses of the protein expression of RUNX2, OCN, and β-actin in the si-NC, si-GDF5-1, and si-GDF5-2 groups after osteogenic induction for 7 days (*n* = 3). Histograms show the quantification of band intensities. β-actin was used for normalization relative to the si-NC group. **f** Images of ALP staining after 7 days of osteogenic differentiation, and ARS staining after 14 days of osteogenic differentiation in the si-NC, si-GDF5-1, and si-GDF5-2 groups (*n* = 3). Histograms show ALP activity and AZR staining quantification by spectrophotometry. **g** Confocal microscopy of RUNX2 with DAPI counterstaining in the si-NC and si-GDF5 groups after osteogenic induction for 7 days (*n* = 3). Scale bars: 20 μm. **h** Relative mRNA expression of ALP, RUNX2, and OCN measured via qRT-PCR in PM and OM on day 7 (*n* = 3). GAPDH was used for normalization. (**p < 0.01; ***p < 0.001). ALP, alkaline phosphatase; GAPDH, glyceraldehyde-3-phosphate dehydrogenase; GDF5, growth differentiation factor 5; hBMSCs, human bone marrow mesenchymal stem cells; PM, proliferation medium; OCN, osteocalcin; OM, osteogenic medium; qRT-PCR, quantitative reverse transcription-polymerase chain reaction; RUNX2, runt-related transcription factor 2

In a previous study, we reported that GDF5 regulates the osteogenic differentiation of human periodontal ligament stem cells [21]. Here, we determined whether GDF5 is involved in the osteogenesis of hBMSCs. Both mRNA and protein expression of GDF5 were increased with osteoinduction in hBMSCs (Fig. 6a, b). Both qRT-PCR and western blotting demonstrated that GDF5 expression was significantly decreased in two GDF5 knockdown groups (Fig. 6c, d). Western blotting analyses demonstrated that the protein expression of RUNX2 and OCN were also downregulated when GDF5 was knocked down in hBMSCs (Fig. 6e). Knockdown of GDF5 decreased ALP staining and activity after 7 days of osteogenic induction (Fig. 6f). The intensity of ARS staining was significantly decreased in the GDF5 knockdown groups after 14 days of osteogenic induction (Fig. 6f). Moreover, immunofluorescence staining of RUNX2 demonstrated that knockdown of GDF5 significantly inhibited osteogenesis (Fig. 6g). In addition, qRT-PCR results showed the decreased mRNA expression of ALP, RUNX2, and OCN when GDF5 was knocked down in both PM and OM conditions (Fig. 6h).

### GDF5 is regulated by YY1/SNHG5/miR-212-3p

**Fig. 7:**
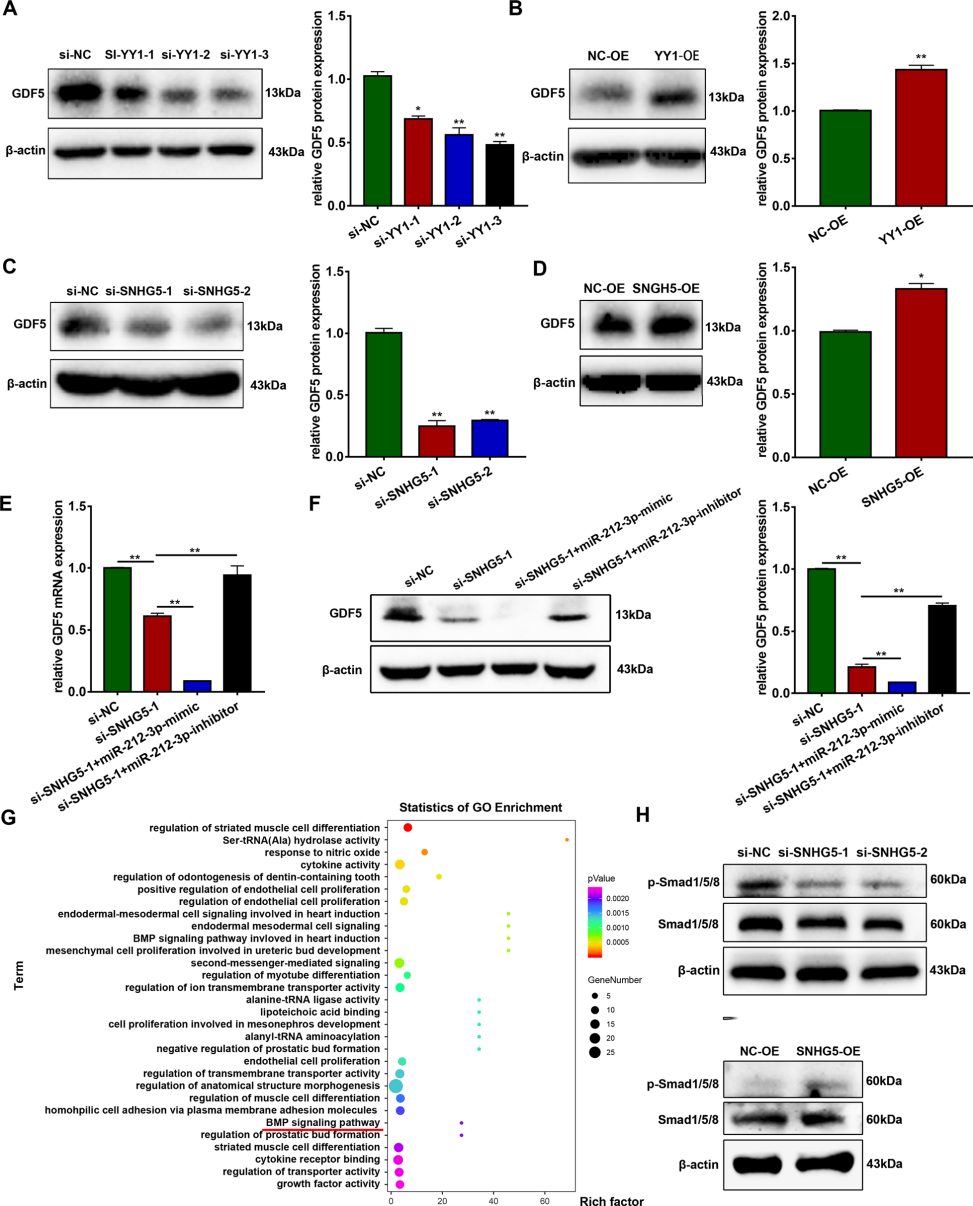
YY1/SNHG5/miR-212-3p/GDF5/Smad axis is involved in the osteogenesis of hBMSCs. **a**–**d** Protein expression of GDF5 and β-actin in groups with YY1 or SNHG5 downregulation or upregulation (*n* = 3). Histograms show the quantification of band intensities. β-actin was used for normalization. **e** Relative mRNA expression of GDF5 measured via qRT-PCR in the si-SNHG5-1, si-SNHG5-1 + miR-212-3p-mimic and si-SNHG5-1 + miR-212-3p-inhibitor transfected hBMSCs (*n* = 3). GAPDH was used for normalization. **f** Protein expression of GDF5 measured via western blotting in the si-SNHG5-1, si-SNHG5-1 + miR-212-3p-mimic and si-SNHG5-1 + miR-212-3p-inhibitor transfected hBMSCs (*n* = 3). Histograms show the quantification of band intensities. β-actin was used for normalization. **g** The top 30 GO term enrichments. **h** Protein expression of p-Smad1/5/8, smad1/5/8, and β-actin in groups with SNHG5 downregulation or upregulation (*n* = 3). (*p < 0.05; **p < 0.01). GAPDH, glyceraldehyde-3-phosphate dehydrogenase; GDF5, growth differentiation factor 5; GO, Gene ontology; hBMSCs, human bone marrow mesenchymal stem cells; qRT-PCR, quantitative reverse transcription-polymerase chain reaction; SNHG5, small nucleolar RNA host gene 5; YY1, Yin Yang 1

To validate whether GDF5 serves as a downstream target of YY1/SNHG5/miR-212-3p, we detected the GDF5 mRNA and protein expression after YY1 or SNHG5 knockdown or overexpression. The results demonstrated that knockdown of YY1 or SNHG5 significantly repressed both mRNA and protein levels of GDF5, whereas overexpression of YY1 or SNHG5 significantly upregulated GDF5 expression (Fig. 7a–d, Additional file 1: Fig. S5G, H). In addition, the rescue assay demonstrated that mRNA and protein expression of GDF5 were significantly downregulated in cells co-transfected with si-SNHG5 and miR-212-3p mimics. By contrast, transfection with si-SNHG5 and miR-212-3p inhibitor synchronous had an opposite effect (Fig. 7e, f). Moreover, knockdown of GDF5 attenuated the enhanced mRNA expression of OCN, ALP, and RUNX2 caused by miR-212-3p knockdown (Additional file 1: Fig. S6A). After knockdown of GDF5 and miR-212-3p, hBMSCs exhibited reduced intensity and activity of ALP and ARS (Additional file 1: Fig. S6B). The protein level of RUNX2 also showed similar results (Additional file 1: Fig. S6C). Together, these data indicate GDF5 acts as a downstream target of YY1/SNHG5/miR-212-3p axis.

### YY1/SNHG5/miR-212-3p/GDF5 promotes the osteogenic differentiation of hBMSCs via accelerated Smad1/5/8 phosphorylation

The TGF-β/Smad signaling pathways participates in the osteogenic process, which is mediated by BMP [28]. Pathway analyses revealed SNHG5 was involved in several signaling pathways including the BMP signaling pathway (Fig. 7g). Therefore, we investigated whether YY1/SNHG5/miR-212-3p/GDF5 promotes osteogenic differentiation of hBMSCs via increasing the phosphorylation of Smad1/5/8. Western blotting analyses were used to detect p-Smad1/5/8 expression in si-SNHG5- and SNHG5-OE-treated hBMSCs. The results demonstrated that p-Smad1/5/8 was significantly decreased in the SNHG5 knockdown groups but was increased in SNHG5 overexpression groups (Fig. 7h).

### SNHG5 and GDF5 promote bone formation in vivo

**Fig. 8:**
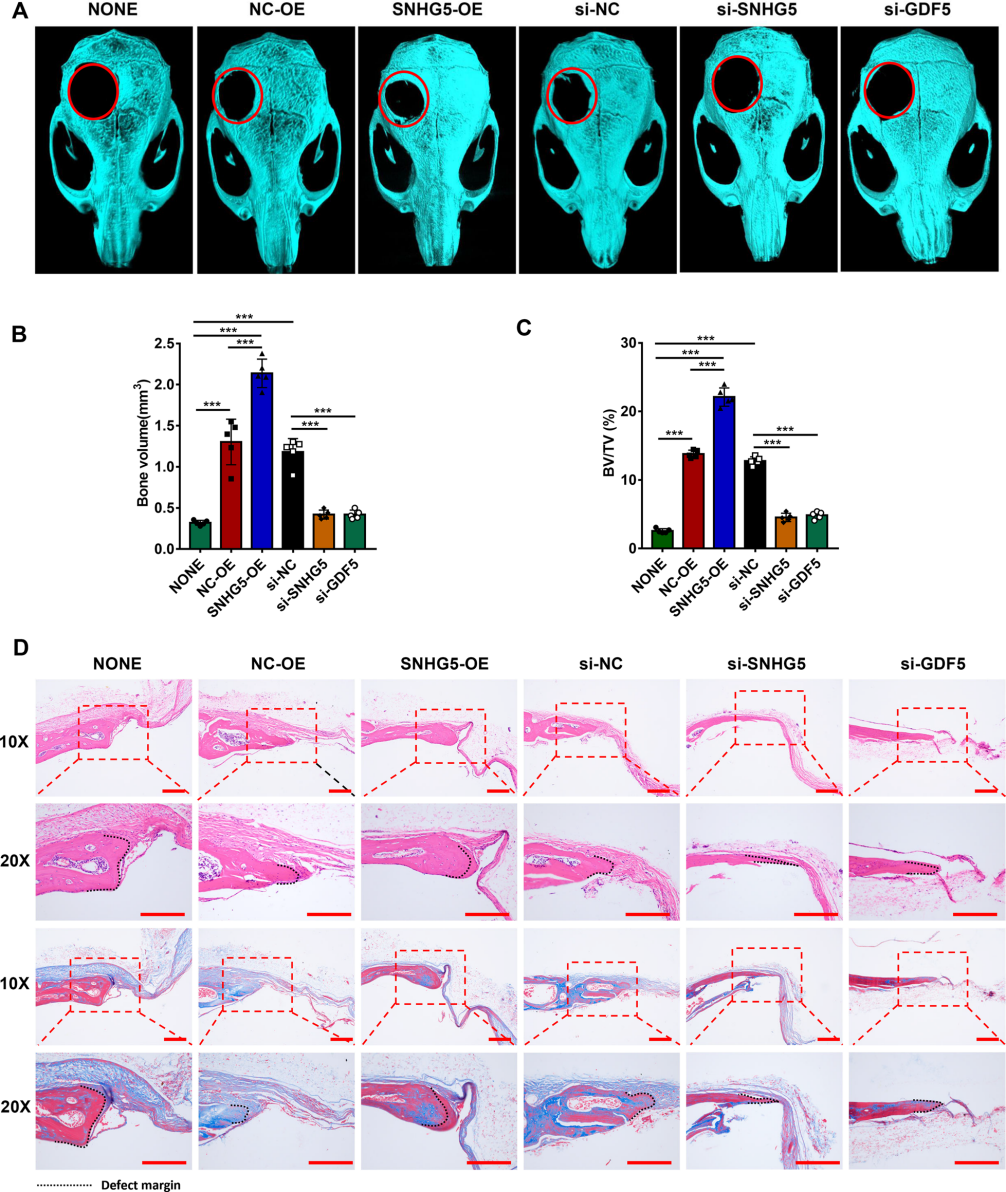
SNHG5 and GDF5 promote bone formation in the critical calvarial defect of mice. **a** Reconstructed 3D micro-CT images of bone formation in each group. **b**, **c** BV and BV/TV analysis of each group at 5 weeks (*n* = 5). **d** H&E staining, Masson’s trichrome staining in each group. Scale bars: 200 μm. (***p < 0.001). BV, bone volume; BV/TV, bone volume/tissue volume; GDF5, growth differentiation factor 5; H&E, hematoxylin and eosin; micro-CT, micro-computed tomography; SNHG5, small nucleolar RNA host gene 5; 3D, three-dimensional

To further explore the role of SNHG5 during osteogenic differentiation of hBMSCs, in vivo animal experiments were conducted. hBMSCs treated with SNHG5-OE, NC-OE, si-SNHG5, si-GDF5, or si-NC were loaded on PLGA scaffold materials and transplanted in the calvarial defects area of nude mice (*n* = 5/group, Additional file 1: Fig. S7A). Skull specimens were harvested 5 weeks later, and 3D reconstructed images demonstrated more new bone formation area and a less defect area in the SNHG5-OE group, and less new bone formation and a larger defect area in the si-SNHG5 and si-GDF5 groups compared to the control group (Fig. 8a). Consistently, BV and BV/TV analyses also indicated more new bone formation in the SNHG5-OE group and less new bone formation in the SNHG5 and GDF5 knockdown groups compared to the control group (Fig. 8b, c, Additional file 1: Table S4). H&E staining and Masson’s trichrome staining showed more newly formed collagen fibers around the defects in the SNHG5 overexpression group, while less collagen formation in the si-SNHG5 and si-GDF5 groups compared to the control group (Fig. 8d). Consistently, immunohistochemistry staining showed that the intensity of the OCN positively stained region in the SNHG5 overexpression group was increased, in the si-SNHG5 and si-GDF5 groups were decreased compared to the control group (Additional file 1: Fig. S7B).

## Discussion

Small nucleolar RNA host gene family members including SNHG5 has been reported regulated osteogenic differentiation [14, 15, 29, 30]. Zheng et al. [14] reported that SNHG5 promoted osteogenic differentiation and apoptosis of hBMSCs via miR-582-5p/RUNX3 feedback loop. However, the underlying mechanisms of SNHG5 in osteogenic differentiation remain largely unknown. In this study, we first found that SNHG5 promoted osteogenic differentiation both in vitro and in vivo, and SNHG5 may serve as a potential target for the treatment of bone loss. SNHG5 also involved in other pathological processes. For example, SNHG5 mediates miR-205-5p, which targets zinc finger E-box-binding homeobox 1 to promote the progression of clear cell renal cell carcinoma [22]. In further clinic use, we should consider about the concentration of SNHG5 to be safely used in the treatment of bone loss.

To further determine the underlying molecular mechanisms by which SNHG5 promotes hBMSCs osteogenesis, we used the JASPAR tool to predict potential binding proteins on the promoter region of SNHG5. The upstream elements of the SNHG5 promoter were analyzed in the region from 3000 bp upstream to the transcriptional start site (TSS) of SNHG5 and the ChIP assays results validated that the vicinity of the 2000 bp region upstream of the SNHG5 TSS contains most of the binding elements of YY1. Li et al. [31] reported that YY1 contributes to angiogenesis via miR-26b/CTGF/VEGFA axis in acute myelogenous by biding SNHG5 promoter region of 2381–2391. The reason for the binding site difference maybe because the different selection of the TSS.

The roles of YY1 in osteogenesis are not fully understood. Only one study has reported that YY1 and HDAC9c can cooperate to increase p38 transcriptional activity and subsequently promote the osteogenic potential of MSCs [32]. Consistently, YY1 promoted osteogenesis both in vitro and in vivo. These results indicate that YY1 promotes the osteogenic differentiation of hBMSCs. miRNAs belonging to the ncRNA family can bind to the complementary sequences in the 3′ UTRs of the target mRNA, and further negatively regulate target gene expression by inhibiting translation or destabilizing the target mRNA. lncRNAs can competitively bind to miRNA and further affect the targets that bind to miRNAs [9]. In this study, both miRNA sequencing and bioinformatics algorithms predicted that SNHG5 contains a binding site for miR-212-3p. miR-212 inhibits osteogenic differentiation via downregulating RUNX2 and repressing the OPG/RANKL pathway [33]. Moreover, miR-212 is upregulated during osteoclast differentiation [34]. We found that SNHG5 negatively regulated miR-212-3p expression, and luciferase reporter assays further confirmed that SNHG5 and miR-212-3p directly interacted with each other, indicating that SNHG5 promotes the osteogenic differentiation of hBMSCs by interacting with miR-212-3p.

GDF5, as a member of the TGF-β superfamily, is the target of several miRNAs including miR-7, miR-132-3p, and miR-615-3p [21, 35, 36]. However, the regulatory mechanism of GDF5 mRNA remains unclear. In our study, we identified GDF5 as a direct target of miR-212-3p via three database analyses combined with microarray analyses. Dual luciferase reporter assays further verified the putative binding of miR-212-3p on GDF5. The mRNA and protein expressions of GDF5 were negatively correlated with miR-212-3p and positively regulated by SNHG5 and YY1. These results indicate that GDF5 acts as a downstream target of YY1/SNHG5/miR-212-3p axis. GDF5 plays critical roles in osteogenic differentiation [37–39]. In accordance with previous studies, GDF5 expression significantly increased with osteoinduction and GDF5 silencing markedly inhibited osteogenic differentiation.

BMPs are members of the TGF-β superfamily and are transduced into cells by the binding of ligands to the type I and type II transmembrane serine/threonine kinase complex. Upon ligand binding, the type II receptor activates the type I receptor by phosphorylation of the type I receptor. The activated type I receptor phosphorylates receptor-activated p-Smad1/5/8, which further forms a complex with Smad4 and together translocate into the nucleus to regulate the transcription of target genes [40]. GDF5 can bind to BMP type I and II receptors to transmit signals into the cytoplasm, leading to activation of p-Smad1/5/8 [41]. In this study, pathway analyses indicated that SNHG5 may participate in the BMP signaling pathway, and we observed that overexpression of SNHG5 activated the BMP pathway while knockdown of SNHG5 inhibited activation of the BMP pathway in hBMSCs. Taken together, these results suggest that the YY1/SNHG5/miR-212-3p/GDF5-related osteogenesis of hBMSCs is modulated through the BMP signaling pathway. However, the mechanisms by which SNHG5 regulates various pathological processes may not be limited to ceRNA mechanisms. Thus, further studies are needed to provide a better understanding of the role of SNHG5 in osteogenesis.

Nevertheless, there are still limitations in this study. We only evaluated the in vivo function of SNHG5 by bone formation assay in nude mice. To further confirm the function of SNHG5, SNHG5 knockout mouse model and more disease models are required. Moreover, the time for critical calvarial defect of mice in our study was 5 weeks, and a longer time for bone formation assay are needed in further experiments. Furthermore, we only used siRNA to knockdown SNHG5 expression in our in vitro experiment, and knockout techniques like CRISPR techniques are needed in our future experiments.

## Conclusion

**Fig. 9:**
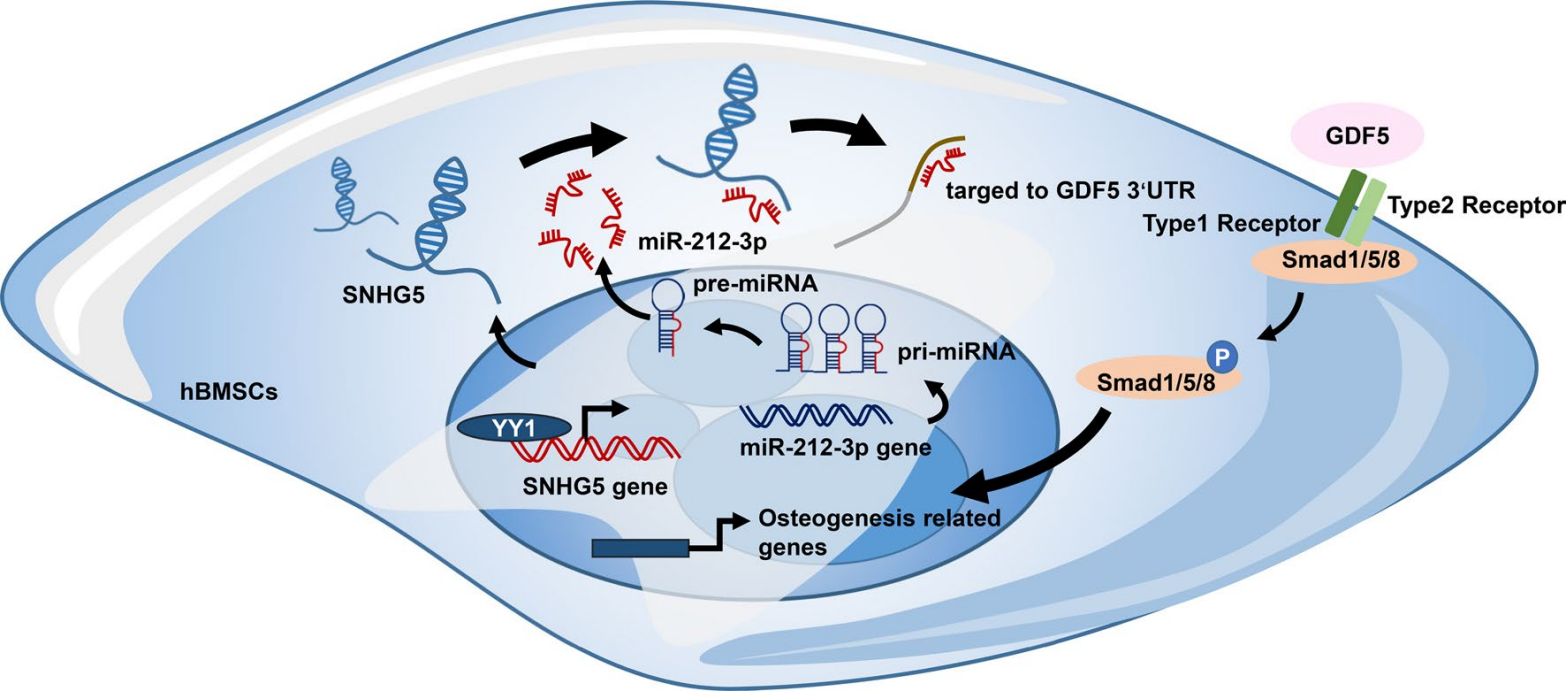
SNHG5 promotes osteogenic differentiation. SNHG5 is regulated by transcription factor YY1 which directly binds to its promoter region to regulate osteogenesis. SNHG5 acts as a miR-212-3p sponge, which further directly targets GDF5 to activate the phosphorylation of Smad1/5/8 to regulate osteogenesis. GDF5, growth differentiation factor 5; OCN, osteocalcin; SNHG5, small nucleolar RNA host gene 5; YY1, Yin Yang 1

In conclusion, SNHG5 promotes osteogenic differentiation of hBMSCs both in vitro and in vivo (Fig. 9). YY1 directly binds to the promoter region of SNHG5 and regulates SNHG5 expression to promote the osteogenic differentiation of hBMSCs. Moreover, SNHG5 sponges miR-212-3p to increase GDF5 and activate p-Smad1/5/8 during osteogenesis. Hence, SNHG5 is a promising target for enhancing the osteogenic potential of hBMSCs and bone regeneration.

## Supplementary Information

**Additional file 1:** Supplementary Materials. **Table S1.** Nucleotide sequence of primers used in qRT-PCR.**Table S2.** The sequences of the RNA oligoribonucleotides.**Table S3.** Primers for ChIP. **Table S4.** Micro-CT results of new bone formation in calvarial defect models. **Figure S1.** Flow cytometry results of cell surface markers (CD90, CD105, CD29, CD34, CD45). **Figure S2.** Overexpression of SNHG5 promotes osteogenic differentiation of hBMSCs. **Figure S3.** Overexpression of YY1 promotes osteogenic differentiation of hBMSCs. **Figure S4.** The location, full-length and secondary structure of SNHG5. **Figure S5.** GDF5 is a target of SNHG5/miR-212-3p axis. **Figure S6.** Knockdown of GDF partially inhibited the osteogenic function of miR-212-3p-inhibitor. **Figure S7.** SNHG5 and GDF5 promote bone formation in the critical calvarial defect of mice.

## Data Availability

The datasets used during the current study has been uploaded to NCBI and the project is PRJNA812856 including 4 accessions numbers (SRR18219241, SRR18219242, SRR18219235, SRR18219236).

## Abbreviations

ALP: Alkaline phosphatase
ARS: Alizarin red S
BCA: Bicinchoninic acid
BMP: Bone morphogenetic protein
BMaSCs: Bone marrow mesenchymal stem cells
bp: Base pairs
BV: Bone volume
BV/TV: Bone volume/tissue volume
ceRNA: Competing endogenous RNA
ChIP: Chromatin immunoprecipitation
DEGs: Differentially expressed genes
DMEM: Dulbecco’s modified Eagle medium
FBS: Fetal bovine serum
FISH: Fluorescence in situ hybridization
GAPDH: Glyceraldehyde-3-phosphate dehydrogenase
GDF5: Growth differentiation factor 5
GO: Gene ontology
hBMSCs: Human bone marrow mesenchymal stem cells
HDAC: Histone deacetylase
H&E: Hematoxylin and eosin
KEGG: Kyoto Encyclopedia of Genes and Genomes database
lncRNAs: Long non-coding RNAs
micro-CT: Micro-computed tomography
miRNAs: MicroRNAs
MaSCs: Mesenchymal stem cells
ncRNAs: Non-coding RNAs
PLGA: Polylactic-co-glycolic acid
OCN: Osteocalcin
OM: Osteogenic medium
PBS: Phosphate-buffered saline
qRT-PCR: Quantitative reverse transcription-polymerase chain reaction
RUNX2: Runt-related transcription factor 2
siRNAs: Small interfering RNA
SNHG5: Small nucleolar RNA host gene 5
TGF-β: Transforming growth factor β
TSS: Transcriptional start site
YY1: Yin Yang 1
α-MEM: α-Modified Eagle medium
2D: Two-dimensional
3D: Three-dimensional
3′ UTR: 3′ Untranslated region
