# Supplementary material for: Long non-coding RNA SNHG5 promotes the osteogenic differentiation of bone marrow mesenchymal stem cells via the miR-212-3p/GDF5/SMAD pathway
Source: Stem Cell Res Ther. 2022 Mar 28;13:130. doi: 10.1186/s13287-022-02781-8 (PMC8962127; doi:10.1186/s13287-022-02781-8)
Supplement: Supplementary file 1 — Additional file 1: Supplementary Materials. Table S1. Nucleotide sequence of primers used in qRT-PCR.Table S2. The sequences of the RNA oligoribonucleotides.Table S3. Primers for ChIP. Table S4. Micro-CT results of new bone formation in calvarial defect models. Figure S1. Flow cytometry results of cell surface markers (CD90, CD105, CD29, CD34, CD45). Figure S2. Overexpression of SNHG5 promotes osteogenic differentiation of hBMSCs. Figure S3. Overexpression of YY1 promotes osteogenic differentiation of hBMSCs. Figure S4. The location, full-length and secondary structure of SNHG5. Figure S5. GDF5 is a target of SNHG5/miR-212-3p axis. Figure S6. Knockdown of GDF partially inhibited the osteogenic function of miR-212-3p-inhibitor. Figure S7. SNHG5 and GDF5 promote bone formation in the critical calvarial defect of mice. [file 13287_2022_2781_MOESM1_ESM.docx]

**Supplementary Materials**

**Table S1. Nucleotide sequence of primers used in qRT-PCR.**

| Gene | Forward primer | Reverse primer |
| --- | --- | --- |
| ALP | ATGGGATGGGTGTCTCCACA | CCACGAAGGGGAACTTGTC |
| RUNX2 | CCGCCTCAGTGATTTAGGGC | GGGTCTGTAATCTGACTCTGTCC |
| OCN | CACTCCTCGCCCTATTGGC | CCCTCCTGCTTGGACACAAAG |
| SNHG5 | GTGGACGAGTAGCCAGTGAAG | GCCTCTATCAATGGGCAGAC |
| YY1 | CCTCTCAGATCCCAAACAACTG | GCCTTTATGAGGGCAAGCTATT |
| GDF5 | GCTGGGAGGTGTTCGACATC | CACGGTCTTATCGTCCTGGC |
| U6 | CTCGCTTCGGCAGCACA | AACGCTTCACGAATTTGCGT |
| MALAT1 | GCTGTGGAGTTCTTAAATATCAACC | TTCTCAATCCTGAAATCCCCTA |
| GAPDH | GGTCACCAGGGCTGCTTTTA | GGATCTCGCTCCTGGAAGATG |

**Table S2. The sequences of the RNA oligoribonucleotides.**

| siRNAs |  |
| --- | --- |
| si-SNHG5-1 | GAUAAUGAAUGUCGAAUGUTT |
| si-SNHG5-2 | GAUGCAAAGAUACACGAAATT |
| si-YY1-1 | GACGACUACAUUGAACAAATTC |
| si-YY1-2 | GCUAGAAUGAAGCCAAGAATT |
| si-YY1-3 | CCUGAAAUCUCACAUCUUATT |
| si-GDF5-1 | GCAACAGCAGCGUGAAGUUTT |
| si-GDF5-2 | CCCAAGAAGGAUGAACCCATT |
| miR-212-3p inhibitor | GGCCGUGACUGGAGACUGUUA |
| miR-212-3p mimic | UAACAGUCUCCAGUCACGGCC |
| miR inhibitor NC | CAGUACUUUUGUGUAGUACAA |
| si-NC | UUCUUCGAACGUGUCACGUTT |

**Table S3 Primers for ChIP.**

| Gene | Forward primer | Reverse primer |
| --- | --- | --- |
| Site1 | CTGTTACCAAGGCTGGAAGG | GCTTGAGCTCAGGAATTGG |
| Site2&3 | GGCCCATCTGATCCTTTTTAC | GTAGAGTCGGGGTTTCACCA |
| Site4 | GGCTTTTGACCAGGTGATGT | CTGCAATCTCGGGATCAAGT |
| Site5 | TGTCGTCTGGATAAAGTATTCAGGG | TCAAACAGCGAACGTGAGGT |
| Site6 | CTCGTAAGAGACGCTTCGCA | CCCTCTCGCACCTATTGGAC |

**Table S4 Micro-CT results of new bone formation in calvarial defect models (n=5).**

|  | BV (mm^3^) | BV/TV (%) |
| --- | --- | --- |
| NONE | 0.32021±0.0268712798355419 | 2.573834±0.293381960699699 |
| NC-OE | 1.303226±0.247638485991172 ^a^ | 13.79224±0.508264964757556 ^a^ |
| SNHG5-OE | 2.137366±0.155075644070886 ^a, b^ | 22.10782±1.1943405400471 ^a, b^ |
| si-NC | 1.180808±0.146939129900787 ^a^ | 12.78222±0.545129846550343 ^a^ |
| si-SNHG5 | 0.420048±0.048131210414864 ^c^ | 4.539788±0.542215358410293 ^c^ |
| si-GDF5 | 0.478546±0.0290923732961063 ^c^ | 4.860168±0.477791255189963 ^c^ |

a, compared with the NONE group, p<0.001; b, compared with the NC-OE group, p<0.001; c, compared with the si-NC group, p<0.001

**Abbreviations:** ALP = alkaline phosphatase; BV = bone volume; BV/TV = bone volume/tissue volume; ChIP = chromatin immunoprecipitation; GAPDH = glyceraldehyde 3-phosphate dehydrogenase; GDF5 = growth differentiation factor 5; MALAT1 = metastasis-associated lung adenocarcinoma transcript 1; micro-CT = micro-computed tomography; OCN = osteocalcin; qRT-PCR = quantitative reverse-transcription polymerase chain reaction; RUNX2 = runt-related transcription factor 2; siRNAs = small interfering RNA; SNHG5 = small nucleolar RNA host gene 5; YY1 = Yin Yang 1.

**Supplementary Figures**


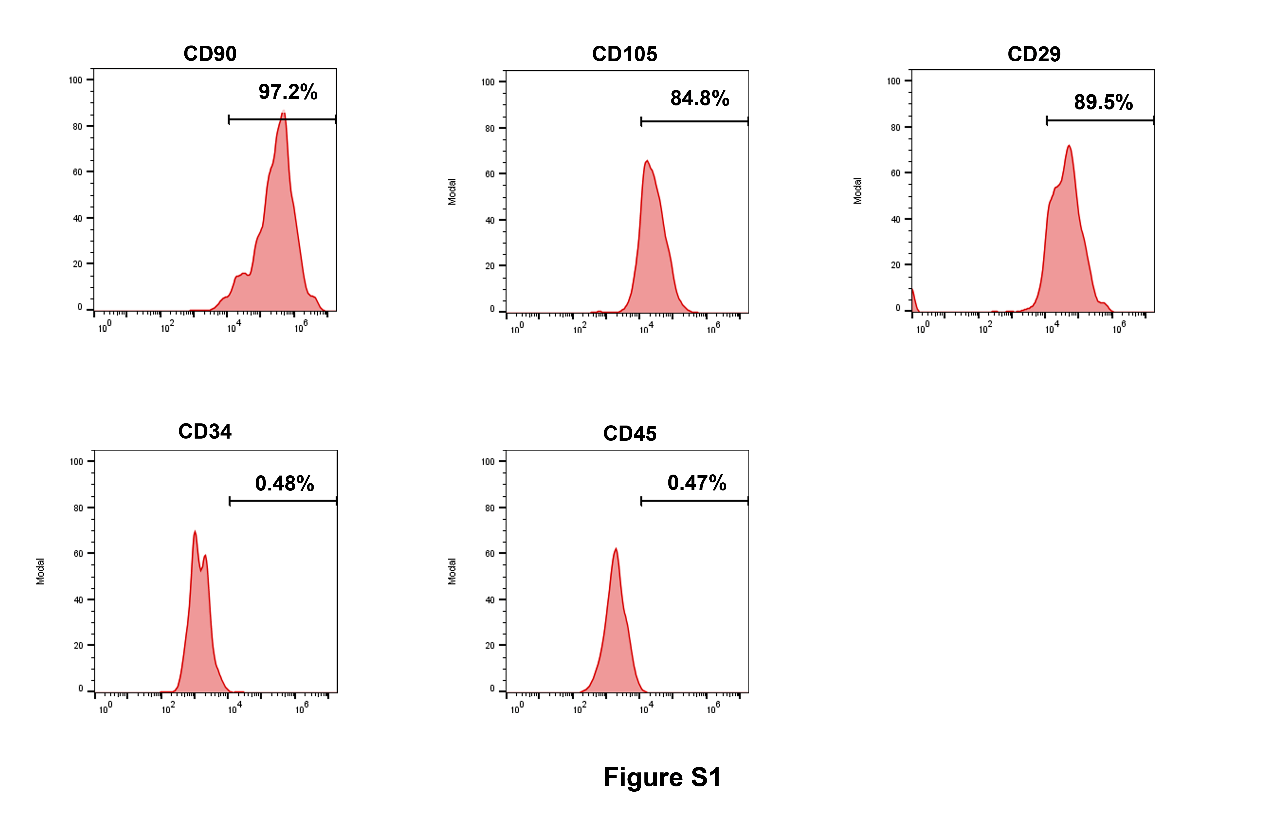


Figure S1. Flow cytometry results of cell surface markers (CD90, CD105, CD29, CD34, CD45).


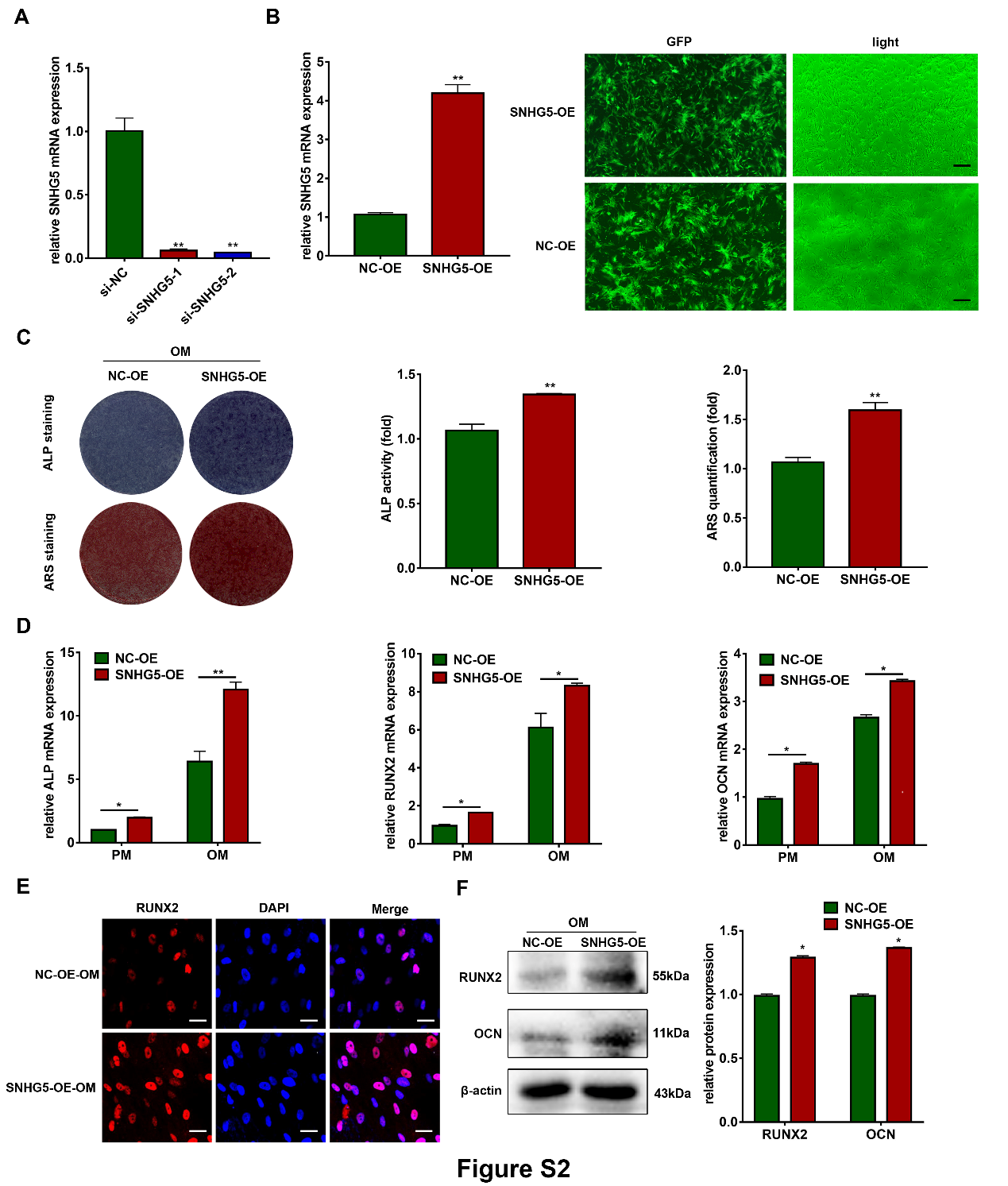


**Figure S2. Overexpression of SNHG5 promotes osteogenic differentiation of hBMSCs.** (A) The efficiency of transient transfection of si-SNHG5 -1, si-SNHG5-2 by qRT-PCR (n=3). GAPDH was used for normalization relative to si-NC group. (B) Fluorescent photomicrographs show the efficiency of lentivirus transduction in hBMSCs expressing SNHG5. Scale bar: 200 μm. qRT-PCR results show the SNHG5 expression after lentivirus transduction compared with the NC-OE groups (n=3). GAPDH was used for normalization relative to the NC-OE groups. (C) Images of ALP staining on day 7 of osteogenic differentiation and ARS staining on day 14 of osteogenic differentiation in the SNHG5-OE, NC-OE groups (n=3). Histograms show ALP activity and ARS staining quantification by spectrophotometry. (D) Relative mRNA expression of ALP, RUNX2, and OCN measured via qRT-PCR in the PM and OM on day 7 (n=3). GAPDH was used for normalization. (E) Confocal microscopy of RUXN2 with DAPI counterstaining of the NC-OE and SNHG5-OE groups after osteogenic induction for 7 days (n=3). Scale bars: 20 μm. (F) Western blotting analyses of the protein expression of RUNX2, OCN and β-actin in the NC-OE, SNHG5-OE groups after osteogenic induction for 7 days (n=3). Histograms show the quantification of band intensities. β-actin was used for normalization relative to the NC-OE groups. (**p*<0.05, ***p*<0.01). Abbreviations: ALP, alkaline phosphatase; ARS, alizarin red S; GAPDH, glyceraldehyde-3-phosphate dehydrogenase; hBMSCs, human bone marrow mesenchymal stem cells; PM, proliferation medium; OCN, osteocalcin; OM, osteogenic medium; qRT-PCR, quantitative reverse transcription-polymerase chain reaction; RUNX2, runt-related transcription factor 2; SNHG5, small nucleolar RNA host gene 5.


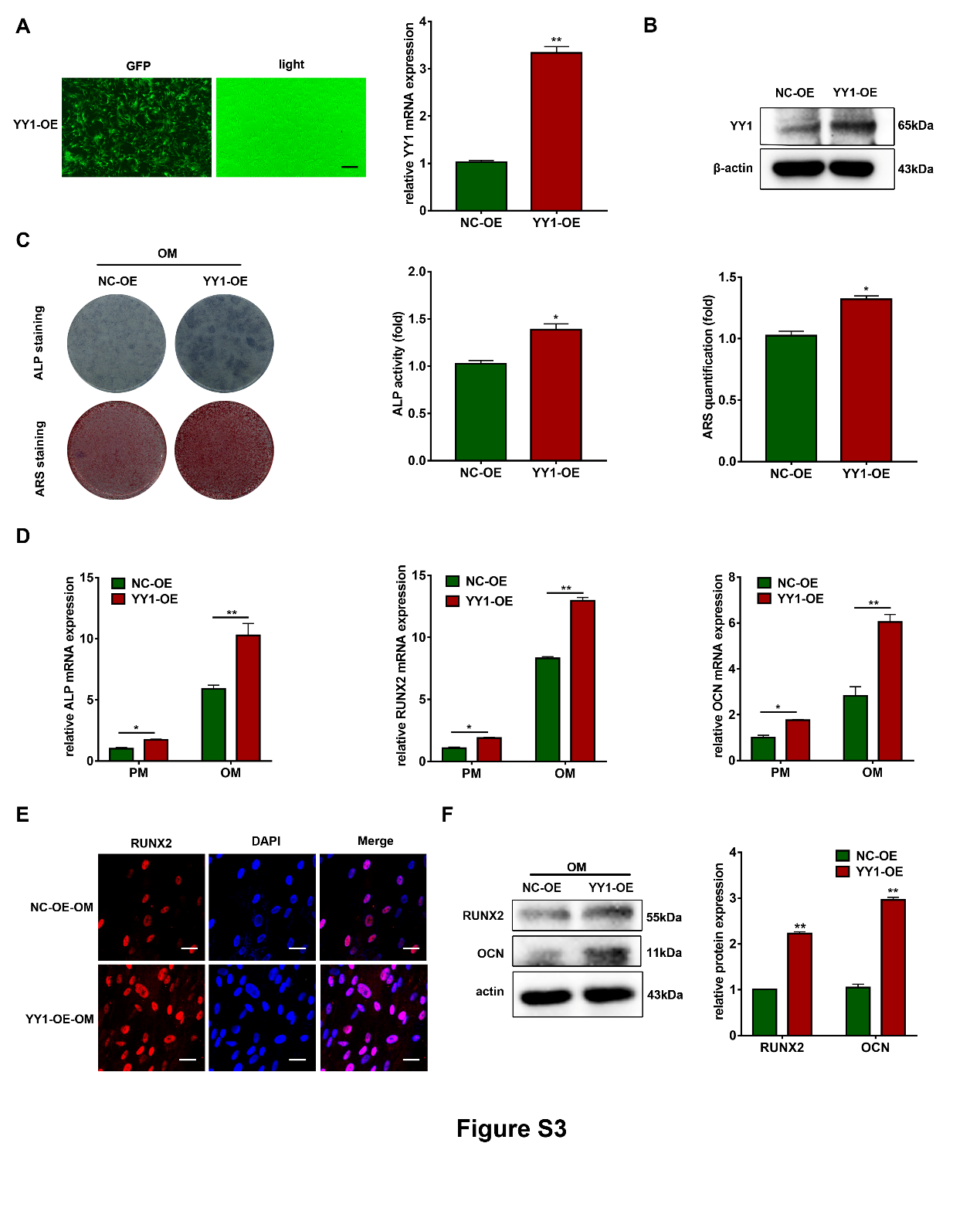


**Figure S3. Overexpression of YY1 promotes osteogenic differentiation of hBMSCs.** (A) Fluorescent photomicrographs show the efficiency of lentivirus transduction in hBMSCs expressing YY1. Scale bar: 200 μm. qRT-PCR results show the mRNA expression of YY1 after lentivirus transduction compared with the NC-OE groups (n=3). GAPDH was used for normalization relative to the NC-OE groups. (B) The efficiency of lentivirus transduction of YY1 by western blotting. Histograms show the quantification of band intensities (n=3). β-actin was used for normalization relative to the NC-OE groups. (C) Images of ALP staining after 7 days of osteogenic differentiation and ARS staining after 14 days of osteogenic differentiation in the YY1-OE, NC-OE groups (n=3). Histograms show ALP activity and ARS staining quantification by spectrophotometry. (D) Relative mRNA expression of ALP, RUNX2 and OCN measured via qRT-PCR in PM and OM on day 7 (n=3). GAPDH was used for normalization. (E) Confocal microscopy of RUXN2 with DAPI counterstaining of the NC-OE, and YY1-OE groups after osteogenic induction for 7 days (n=3). Scale bars: 20 μm. (F) Western blotting of the protein expression of RUNX2, OCN and β-actin in the NC-OE, YY1-OE groups after osteogenic induction for 7 days (n=3). Histograms show the quantification of band intensities. β-actin was used for normalization relative to the NC-OE group. (**p*<0.05, ***p*<0.01). Abbreviations: ALP, alkaline phosphatase; ARS, alizarin red S; ChIP, Chromatin immunoprecipitation; GAPDH, glyceraldehyde-3-phosphate dehydrogenase; hBMSCs, human bone marrow mesenchymal stem cells; PM, proliferation medium; OCN, osteocalcin; OM, osteogenic medium; qRT-PCR, quantitative reverse transcription-polymerase chain reaction; RUNX2, runt-related transcription factor 2; YY1, Yin Yang 1.


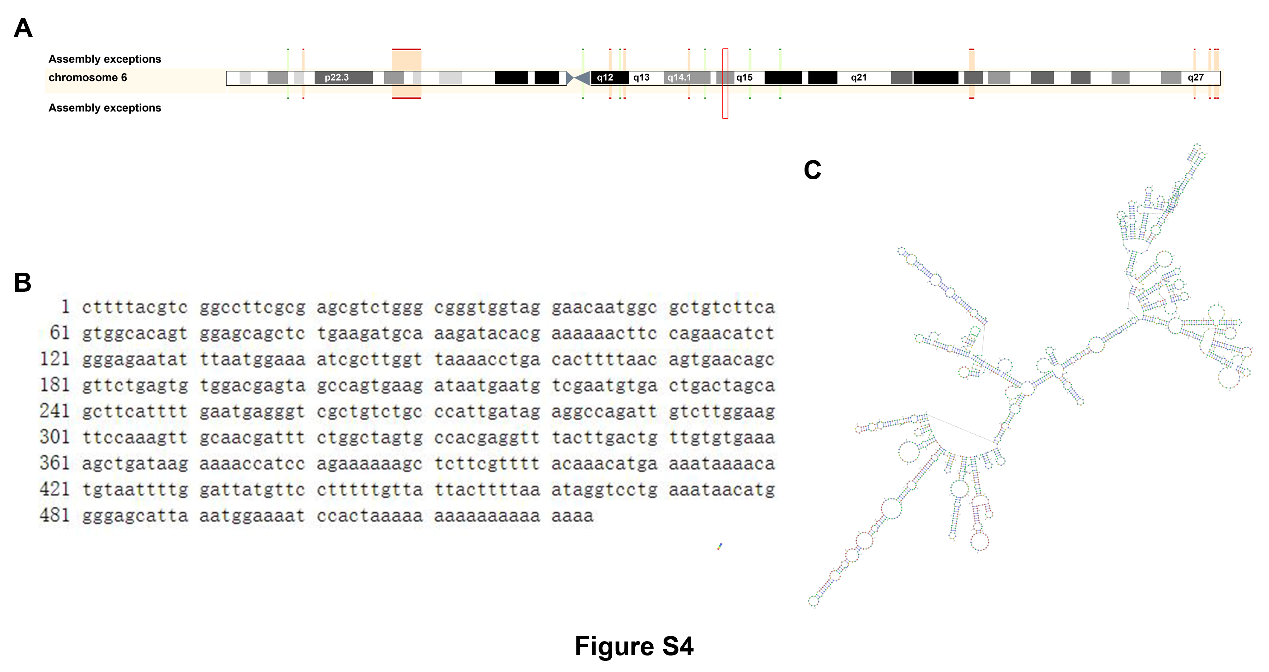


**Figure S4.** (A) SNHG5 Gene in genomic location (6q14) according to GeneLoc; bands according to Ensembl. (B) The full-length sequence of SNHG5 from the GeneCards website. (C) Secondary structure of SNHG5 from GeneCards website. Abbreviations: SNHG5, small nucleolar RNA host gene 5.


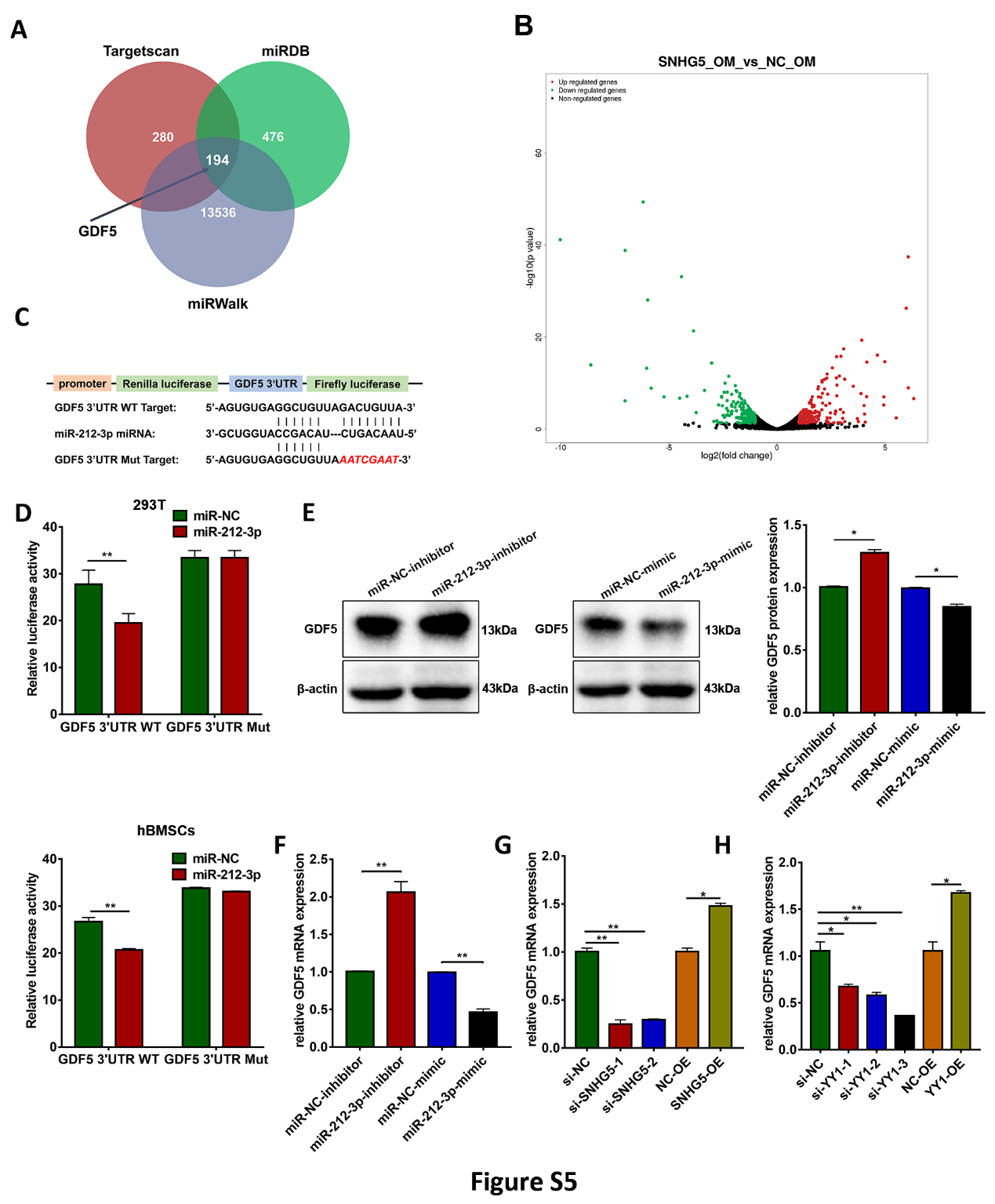


**Figure. S5.** **GDF5 is a target of SNHG5/miR-212-3p axis.** (A) Venn diagrams show the number of potential genes targeting miR-212-3p (including the GDF5 gene). The potential genes were predicted by three databases: TargetScan, miRWalk and miRDB. (B) Volcano plot show dysregulated genes in SNHG5 knockdown hBMSCs 7 days after osteoinduction compared with the control group. (C) Schematic diagram of the miR-212-3p putative binding site in GDF5 3’ UTR WT and GDF5 3’ UTR Mut. (D) Dual luciferase reporter assay validates the interaction between miR-212-3p and GDF5 in 294T cells and hBMSCs (n=3). (E) Relative protein expression of GDF5 and β-actin in miR-NC-inhibitor, miR-212-3p-inhibitor, miR-NC-mimic, and miR-212-3p-mimic groups (n=3). The histograms show the quantification of band intensities. β-actin was used for normalization. (F) Relative mRNA expression of GDF5 after downregulation or upregulation of miR-212-3p by qRT-PCR (n=3). GAPDH was used for normalization. (G, H) Relative mRNA expression of GDF5 measured by qRT-PCR with SNHG5 or YY1 knockdown or overexpression (n=3). GAPDH was used for normalization. (**p*<0.05, ***p*<0.01). Abbreviations: GAPDH, glyceraldehyde-3-phosphate dehydrogenase; GDF5, growth differentiation factor 5; hBMSCs, human bone marrow mesenchymal stem cells; qRT-PCR, quantitative reverse transcription-polymerase chain reaction; SNHG5, small nucleolar RNA host gene 5; YY1, Yin Yang 1.


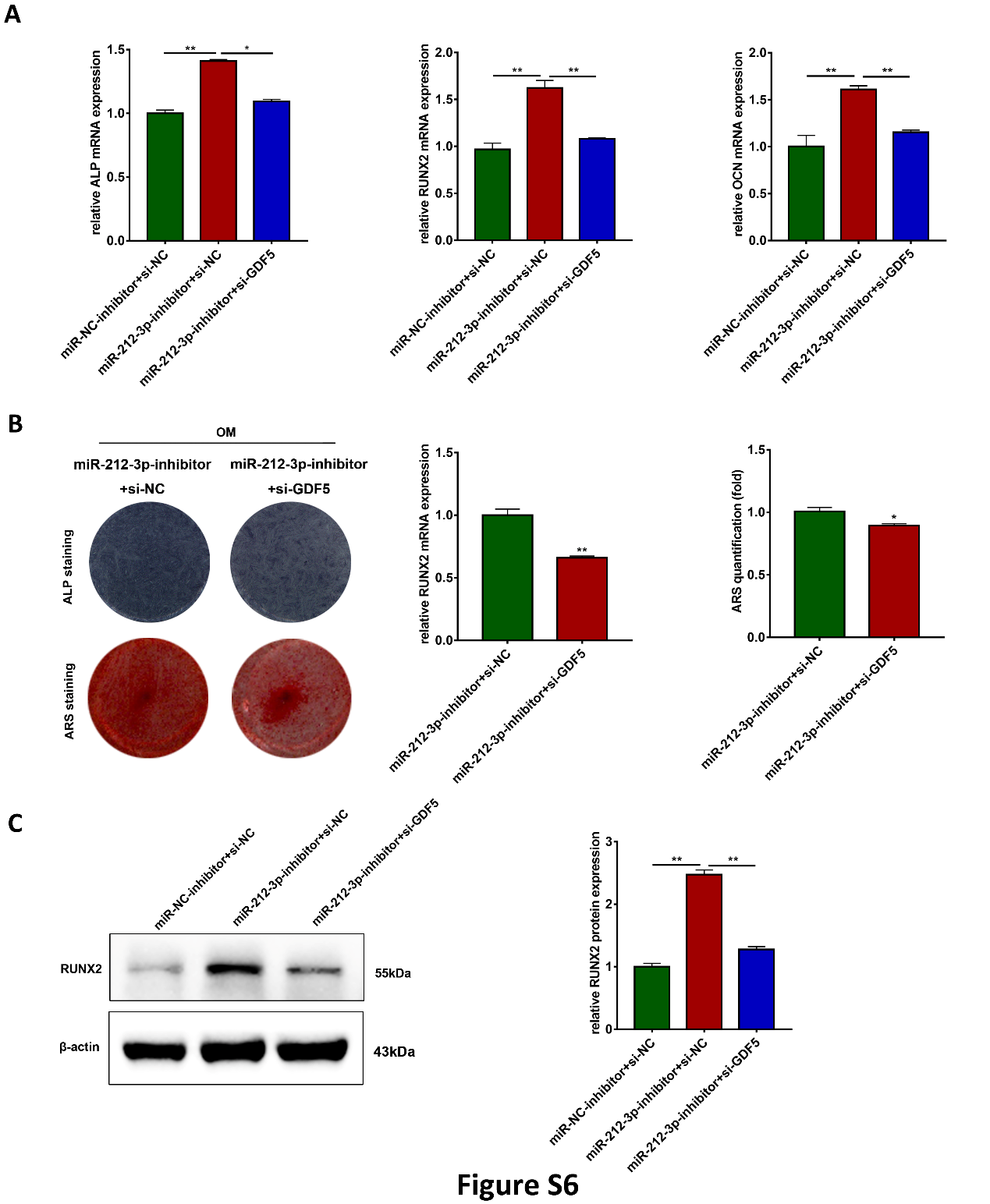


**Figure S6. Knockdown of GDF partially inhibited the osteogenic function of miR-212-3p-inhibitor.** (A) Relative mRNA expression of ALP, RUNX2, and OCN measured via qRT-PCR in PM conditions (n=3). GAPDH was used for normalization. (B) Images of ALP staining after 7 days of osteogenic differentiation, and ARS staining after 14 days of osteogenic differentiation in the miR-212-3p-inhibitor+si-NC, miR-212-3p-inhibitor+si-GDF5 groups (n=3). Histograms show ALP activity and AZR staining quantification by spectrophotometry. (C) Western blotting analyses of the protein expression of RUNX2 and β-actin in the miR-NC-inhibitor+si-NC, miR-212-3p-inhibitor+si-NC, miR-212-3p-inhibitor+si-GDF5 groups after osteogenic induction for 7 days (n=3). Histograms show the quantification of band intensities. β-actin was used for normalization relative to the si-NC group. (*p<0.05, **p<0.01). Abbreviations: ALP, alkaline phosphatase; GAPDH, glyceraldehyde-3-phosphate dehydrogenase; GDF5, growth differentiation factor 5; hBMSCs, human bone marrow mesenchymal stem cells; PM, proliferation medium; OCN, osteocalcin; qRT-PCR, quantitative reverse transcription-polymerase chain reaction; RUNX2, runt-related transcription factor 2.


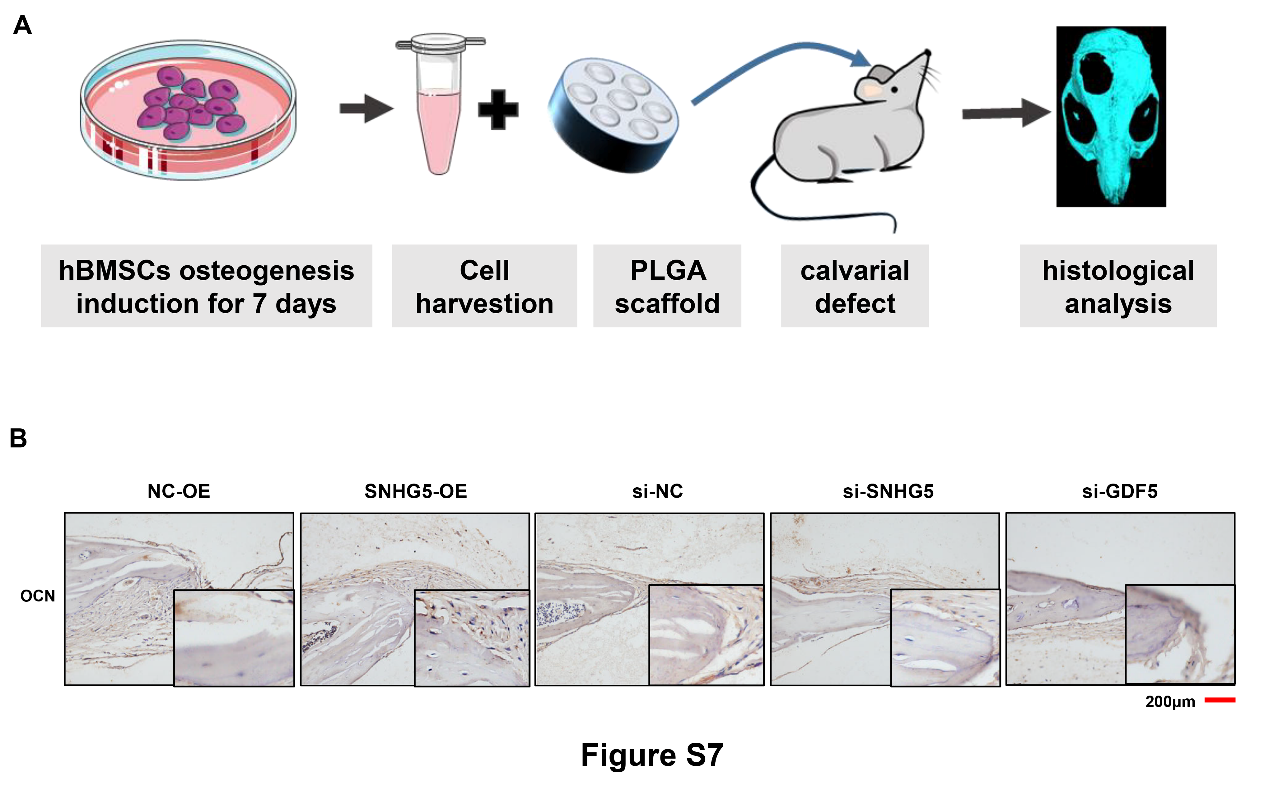


**Figure S7. SNHG5 and GDF5 promote bone formation in the critical calvarial defect of mice.** (A) Schematic diagram illustrates the experimental procedure. (B) Immunohistochemical staining of OCN in each group. Scale bars: 200 μm. Abbreviations: GDF5, growth differentiation factor 5; OCN, osteocalcin; SNHG5, small nucleolar RNA host gene 5.
